# The potential of non-native tree species to provide major ecosystem services in Austrian forests

**DOI:** 10.3389/fpls.2024.1402601

**Published:** 2024-07-01

**Authors:** Julia Konic, Carina Heiling, Elena Haeler, Debojyoti Chakraborty, Katharina Lapin, Silvio Schueler

**Affiliations:** ^1^ Department for Forest Growth, Silviculture & Genetics, Austrian Research Centre for Forests (BFW), Vienna, Austria; ^2^ Department of Forest Biodiversity and Nature Conservation, Austrian Research Centre for Forests (BFW), Vienna, Austria

**Keywords:** forestry, NNT, timber yield, tree species richness, protection forest, avalanche, rockfall

## Abstract

Forestry is facing an unprecedented challenging time. Due to climate change, major tree species, which until recently fulfilled major ecosystem services, are being lost and it is often unclear if forest conversion with other native or non-native tree species (NNT) are able to maintain or restore the endangered ecosystem services. Using data from the Austrian Forest Inventory, we analysed the current and future (2081-2100, RCP 4.5 and RCP 8.5) productivity of forests, as well as their protective function (avalanches and rockfall). Five different species change scenarios were considered for the replacement of a tree species failing in the future. We used seven native tree species (*Picea abies, Abies alba, Pinus sylvestris, Larix decidua, Fagus sylvatica, Quercus robur* and *Quercus petraea*) and nine NNT (*Pseudotsuga menziesii, Abies grandis, Thuja plicata, Pinus radiata, Pinus contorta, Robinia pseudoacacia, Quercus rubra*, *Fraxinus pennsylvanic*a and *Juglans nigra*). The results show that no adaptation would lead to a loss of productivity and a decrease in tree species richness. The combined use of native and NNT is more favorable than purely using native species in terms of productivity and tree species richness. The impact of the different species change scenarios can vary greatly between the different environmental zones of Austria (Alpine south, Continental and Pannonian). The Pannonian zone would benefit from the use of NNT in terms of timber production. For the protection against avalanches or rockfall in alpine regions, NNT would not be an advantage, and it is more important if broadleaved or coniferous trees are used. Depending on whether timber production, protective function or tree species richness are considered, different tree species or species change scenarios can be recommended. Especially in protective forests, other aspects are essential compared to commercial forests. Our results provide a basis for forest owners/managers in three European environmental zones to make decisions on a sustainable selection of tree species to plant in the face of climate change.

## 1 Introduction

In recent decades, the effects of climate change on Austrian forests have become increasingly apparent (Strauss et al., 2013; Olefs et al., 2021). The rise in temperature, the shift in precipitation patterns and the increased occurrence of extreme weather events are significantly affecting the vitality and composition of forest ecosystems (Lindner et al., 2014; Seidl et al., 2014; Choat et al., 2018). The environmental impacts also extend beyond forests to the complex web of ecosystem services they provide (Cudlín et al., 2013; Bugmann et al., 2015; Mina et al., 2017; Albrich et al., 2018). These services encompass a spectrum of essential benefits to humanity, such as wood production (Verkerk et al., 2014) and water supply (Brockerhoff et al., 2017) to climate regulation and recreation (Hassan et al., 2005). Ecosystem services can be classified into four main types: provisioning, regulating, cultural and supporting (Millennium Ecosystem Assessment, 2005; La Notte et al., 2017). Forests provide a central function in the fulfilment of these services, and alterations to the forest ecosystem can have profound effects on society as a whole (IPCC, 2019). For example, the effects of climate change have a significant impact on the availability of natural resources that are closely linked to forests (Lindner et al., 2010). Changes in forest structure, more frequent calamities and increasing pest infestations can severely hamper the timber industry (e.g. Schuck and Schelhaas, 2013; Steyrer et al., 2020; Hlásny et al., 2021; Panzavolta et al., 2021; Van Hensbergen and Cedergren, 2021). Thus, the consequences go beyond the economic sphere and affect the livelihoods of many people working in the sector. Furthermore, trees act as important carbon sinks, absorbing carbon dioxide from the atmosphere and storing it in the form of carbon compounds as they grow (Lorenz and Lal, 2010). An uncontrolled forest decline will release stored carbon into the atmosphere, increasing greenhouse gas emissions which accelerate climate change (Kurz et al., 2008). This creates a feedback loop that can intensify the climate crisis (IPCC, 2019).

**Figure 1:**
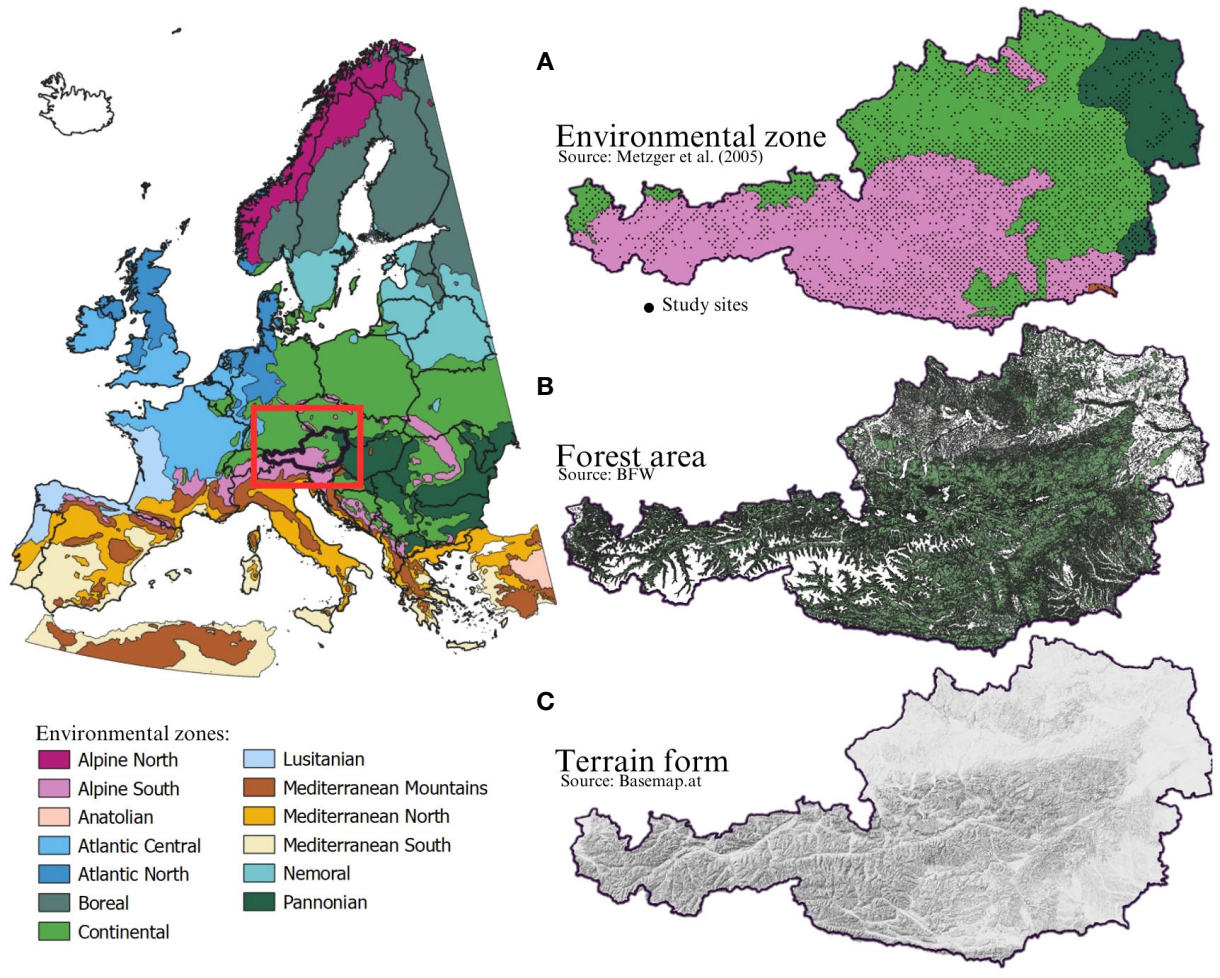
Environmental zones of Europe according to Metzger et al. (2005). **(A)** shows the environmental zones of Austria and the study sites in detail; Alpine south (1423 study sites), Continental zone (1448 study sites), Pannonian zone (150 study sites) and Mediterranean Mountains (3 study sites). **(B)** shows the forest area of Austria and **(C)** shows the terrain of Austria according to Basemap.at.

According to Metzger et al. (2005) Austria belongs to 4 of the 13 environmental zones in Europe: the Alpine south, the Continental zone, the Pannonian zone and the Mediterranean Mountains (**Figure 1**). The Alpine south (Bogers, 2013a) spans high, medium, and low mountains in Central and Southern Europe. It includes ranges from the Alpine orogenic belt (e.g., Pyrénées, Alps, Carpathians, Tatr, Balkan Mountains) and Hercynian Europe (e.g., Schwarzwald, Thüringer Wald, Harz, Erzgebirge, Sudety). These areas feature classic Alpine landscapes with deep valleys and permanent snow on high peaks, lower mountains and uplands. Climate and vegetation vary significantly by region and slope orientation. The Continental zone (Bogers, 2013b) covers the plains and lowlands of Central and Eastern Europe, and the uplands and low mountains of the Balkan Peninsula, from the Ardennes to Ukraine. It has a continental climate with significant seasonal temperature variations and summer peak precipitation. Potential vegetation includes deciduous forests in the west and mixed and coniferous forests centrally. Beech forests are prominent. The fertile soil supports extensive agriculture, while forestry is common in hills and mountains. The Pannonian zone (Bogers, 2013c), encompasses lowlands, valleys, and mountain peripheries in the Middle- and Lower-Danube Plains and the Black Sea Lowland, including part of the Rhine Valley. It features flat terrain, a dry continental climate with summer precipitation peaks, and steppe-like vegetation. Potential vegetation includes mixed Acer and Turkish oak forests and steppe grasslands (*Stipa* sp.). Historically, the area focused on grassland farming, now largely converted to crops. The effects of climate change on Austria’s forests are becoming more apparent, although they vary in intensity and characteristics depending on the environmental zone. Given that only a very small proportion of Austria (3 out of 3024 study sites) is situated within the Mediterranean mountain zone, we excluded this zone from the present study.

Past studies have shown a decrease in the productivity due to climate change in the Continental and Pannonian zone resulting in reduced increment and hence reduced carbon sequestration from the atmosphere (Anderegg et al., 2015; Ols et al., 2022). Meanwhile, in Alpine South, the crisis caused by bark beetles and windstorms is reaching an unprecedented level (Steyrer et al., 2022). Bark beetle infestations damage the protective function of forest ecosystems (Seidl et al., 2009) threatening settlements and infrastructure in alpine regions.

Steep alpine terrains, which are frequently occurring in the Alpine South zone in Austria (**Figure 1C**) are susceptible to hazards like avalanches, rockfalls, and mudflows (Fischer et al., 2012). Alpine forests (**Figure 1B**) play a crucial role as natural barriers, safeguarding settlements and infrastructure by slowing down and containing avalanches (Bebi et al., 2009; Védrine et al., 2022). The intricate root network of these forests further prevents soil erosion, offering protection against rockfall and mudflows (Dorren and Schwarz, 2016). Additionally, the forest canopy efficiently absorbs precipitation and releases it in a controlled manner, maintaining the soil’s structural integrity and reducing the risk of rock masses sliding or soil erosion (Sidle and Ochiai, 2006). Therefore, the vegetation in these mountain forests serves as more than just a physical barrier. It also acts as an absorption layer that helps to better regulate snow and rock masses. This dual functionality not only reduces the risk of damage, but also emphasizes the ability of forests to mitigate the potential impact of environmental hazards in nature (Perzl et al., 2022). For this reason, the Austrian government launched an ‘Action Programme Protective Forest’ a few years ago (BML, 2024). The main goal of this action programme is the maintenance, preservation, and research of these important forests.

A critical challenge for Austrian forests is the decreasing climate suitability of native tree species and tree communities (Thuiller, 2003; Buras and Menzel, 2019; Chakraborty et al., 2021), due to the rapid changes in local climate conditions. The ongoing and predicted local species diebacks are expected to result in shifts in the overall forest structure (Walentowski et al., 2017; Menezes-Silva et al., 2019). This could not only lead to the loss of economically important tree species, but also a decline in present forest biodiversity. Certain species may struggle to find suitable habitats or cannot adapt quickly enough to the altered conditions. This problem is further exacerbated by the long lifespan of trees, which clashes with rapidly changing climatic conditions (Jandl et al., 2019). One way to counteract the loss of diversity, timber production and carbon sequestration potential in Austria’s forests under future climate could be the promote of non-native tree species (NNT). NNT originating from regions that already have similar climatic conditions to those expected in Austrian forests may have characteristics and local adaptations that will allow them to survive and eventually perform better than many native tree species (Brang et al., 2016).

Many fast-growing, stress-tolerant, or aesthetically pleasing tree species have been broadly planted outside their native range (Dickie et al., 2014). The motivation for their introduction differs widely and may include the planting of non-native conifers for timber and pulp production (Brundu and Richardson, 2016), the reclamation of land and stabilization of sand dunes (Evans, 2009; Griffin et al., 2011), the deployment of legume trees (e.g. *Acacia or Gleditsia*) to combat desertification and provide resources in arid regions (Witt, 2017), or the use of ornamental trees to enhance environments for rural and urban populations (e.g. shade) (Dickie et al., 2014). Previous research has underscored the intricate and situation-dependent effects of NNT on ecosystem services. Castro-Díez et al. (2019) showed that non-native tree species are more likely to increase than decrease the ecosystem services attributed to NNT. Especially regulating ecosystem services, such as climate regulation, erosion control, and soil fertility, can benefit from the use of NNT (Castro-Díez et al., 2021). Schlaepfer et al. (2020) further emphasized the significance of non-native trees in urban environments, where they contribute to biodiversity and various ecosystem services, particularly those of cultural importance. Expanding on this perspective, Catterall (2016) highlighted the involvement of NNT in large-scale forest regeneration efforts and the necessity of considering their functional roles alongside their biogeographical origins. NNT can further support the regeneration of native species by improving local site conditions (Richardson et al., 2000). However, the use of NNT also poses several risks and concerns, including the invasive potential of some species, changes in native biodiversity, and changes in the provision of ecosystem services (Vilà et al., 2011; Pötzelsberger et al., 2020). Hence, it is required to carefully evaluate the potential invasiveness of NNT species before introducing them to new ecosystems (e.g. Bindewald et al., 2021).

Therefore, the objectives of this study were (1) to investigate the influence of climate change on the composition of native tree species and their ecosystem services, (2) to assess the role of NNT in facilitating forest adaptation to climate change to secure ecosystem services, and (3) to compare the impact of adaptation scenarios without NNT with those that include NNT.

## 2 Materials and methods

### 2.1 Data and distribution models

To analyze the future composition of tree species and their contribution to different ecosystem services, we based our study on seven major native tree species in Austria: *Abies alba, Fagus sylvatica, Larix decidua, Picea abies, Pinus sylvestris, Quercus robur* and *Quercus petraea*, which cover in total around 84% of Austria’s managed and stocked forests (www.waldinventur.at). Since the two closely related white oak species share a similar distribution area in Austria, and are not discriminated in national forest inventory data, these two species were treated together as *Quercus* spp. Data from the occurrence and productivity (average increment) of these seven species was obtained from the Austrian forest inventory (period 2007–2009), which encompasses the entire federal territory and comprises 3141 permanent survey sites, each consisting of up to 4 plots.

To evaluate the possible contribution of NNT to forest ecosystem services, nine NNT were evaluated for their climatic suitability and their potential contribution to ecosystem services in Austria under climate change. These nine species were selected based on the following criteria: 1) species that are already well known, tested or established in Austria as forest species for forestry use (*Abies grandis, Juglans nigra, Pseudotsuga menziesii, Quercus rubra, Thuja plicata*), 2) species introduced to Austria as ornamental plants which are, however, spreading in forests due to their invasive characteristics (*Fraxinus pennsylvanica, Robinia pseudoacacia*), 3) species with worldwide importance as timber species but not yet present in Austrian forests (*Pinus contorta, Pinus radiata*), 4) availability of sufficient data to establish species distribution models and productivity as basis for an estimation of ecosystem services (all nine NNT). The level of knowledge in terms of their ecological characteristics, their invasive potential and cultivation in Central Europe varies widely for these nine species (**Supplementary Table S1**).

To assess the suitability of the seven native tree species and their contribution for delivering ecosystem services in future forests, we used species distribution models (SDMs) that have been established previously by Chakraborty et al. (2021). As similar models were not available for the NNT of interest, the current and potential future distribution (probability of occurrence) of the nine NNT was estimated following the same modelling approach as for native species (Chakraborty et al., 2021). To reduce the uncertainty arising due to modelling algorithm, input data and assumptions therein a multi model ensemble approach was selected. This ensemble model included 10 different SDMs implemented through the Biomod2 platform (Thuiller et al., 2013). Assumptions for each models in the Biomod2 are also detailed in the **Supplementary Material** (**Supplementary Table S3**).

10 modeling algorithms were selected: GLM (Generalized Linear Models), GAM (Generalized Additive Models), GBM (Generalized Boosted regression Models), CTA (Classification Tree Analysis), ANN (Artificial Neural Networks), SRE (Surface Range Envelop or BIOCLIM), FDA (Flexible Discriminant Analysis), MARS (Multivariate Adaptive Regression Spline), RF (Random Forest for classification and regression), and MAXENT. The SDMs were calibrated with the observed occurrence and bioclimatic variables of the NNT in their natural range and their introduced range in Europe (**Supplementary Table S2**). The bioclimatic variables used to calibrate the SDMs were obtained from Worldclim database v2.0 (Fick and Hijmans, 2017). The occurrence data for the nine NNT at their native and introduced range in Europe was obtained from various sources such as national forest inventories, global biodiversity facilities, etc. as collected by the EU-COST Action NNEXT (Brus et al., 2019). The occurrence dataset for each target species was partitioned by splitting into 75% for model training and 25% for model evaluation. The dataset includes a total of 754, 413 occurrence records of the target species (i.e. presence locations). The assumptions and detail of input data are mentioned in **Table S3** in the **Supplementary material**. With this approach the potential distribution of each species was estimated by each of the 10 SDMs in biomod2 in historical climate (1961–1990) and two future climate scenarios, RCP 4.5 and 8.5 for the periods 2041–2060, 2061–2080, and 2081–2100. Potential distribution in future scenarios is based on the mean of the 13 RCM projections available in Worldclim database v2.0 (Hijmans et al., 2005). Predicted probabilities from the individual models for each target species were ensembled as a consensus model which combined the median probability over the selected models with the true skill statistics threshold (TSS > 0.7). The predicted potential distributions for historical and future climates are available as GeoTiff rasters with a spatial resolution of 30 arcsec, which is roughly equivalent to 1x1 km or lower depending on latitude, in WGS 84 projection from ZENODO (Chakraborty et al., 2024). The **Supplementary Material** provides a more detailed account of the SDMs.

For the actual occurrence and species share in Austrian forests, the data from the four inventory plots per survey site were aggregated to the centroids of the sites. The mean values were used to aggregate the values for the tree species data, while the median was used to aggregate the slope and altitude of the respective survey site. This approach was taken to increase accuracy and precision. If none of the 15 tree species (**Table 1**) was currently present at the centroid, then this centroid was neglected for further calculations. Out of a total of 3141 centroids, 117 were thus removed from the dataset. So, the models were applied to 3024 centroids. The calculations and graphical representations were executed in Python 3.11, using essential libraries such as Pandas, NumPy, GeoPandas, Rasterio, Matplotlib and Pickle.

**Table 1:** List of native and non-native species considered as main stand-forming tree species and species characteristics used in the present analysis.

Species	Native	Mean model cut-off^1^ [%]	Annual Increment mean value [m³ over bark]	Annual Increment standardized^2^	Resource [Increment value]
*Abies alba*	Yes	46.4	15.59	1.26	ÖWI^3^-Data (BFW, 2022)
*Fagus sylvatica*	Yes	50.2	8.17	0.66	ÖWI-Data (BFW, 2022)
*Larix decidua*	Yes	52.1	7.76	0.62	ÖWI-Data (BFW, 2022)
*Picea abies*	Yes	56.5	11.77	0.95	ÖWI-Data (BFW, 2022)
*Pinus sylvestris*	Yes	63.1	7.47	0.6	ÖWI-Data (BFW, 2022)
*Quercus* spp.	Yes	48.3	9.64	0.78	ÖWI-Data (BFW, 2022)
*Abies grandis*	No	65.0	31.85	2.56	Vor et al. (2015)
*Fraxinus pennsylvanica*	No	56.7	3.65	0.29	Burns and Honkala (1990)
*Juglans nigra*	No	62.8	9.57	0.77	Ehring et al. (2019)
*Pinus contorta*	No	69.1	3.85	0.31	Alexander and Edminster (1981), DeSpain (1973)
*Pinus radiata*	No	53.2	22.5	1.81	Wagner (2012)
*Pseudotsuga menziesii*	No	46.4	19.08	1.54	Klädtke (2016); BFW (2017a); BFW (2018),
*Quercus rubra*	No	59.2	11.15	0.9	Engel (2001)
*Robinia pseudoacacia*	No	66.9	10.68	0.86	Engel (2001); Vor et al. (2015); de Avila and Albrecht (2017), Rédei (1998)
*Thuja plicata*	No	55.5	16.38	1.32	Lockow (2002); Panka (2014); BFW (2017b)

^1^Threshold value for modelling climate suitability, below which a value is assessed as zero, as outlined by Chakraborty et al. (2021).

^2^Standardized annual increment value of 1, representing the mean across all tree species, results in an increment of 12.6 m³ over bark per year/hectare.

^3^ÖWI = Austrian Forest Inventory – not publicly accessible data set.

### 2.2 Species occurrence probability

To specify the potential tree species composition at the inventory centroids, the probability of occurrence, respectively the suitability under past (1961–1990) and future climate (2081–2100, RCP 4.5 and 8.5) were retrieved from the species distribution models of all native and non-native species. The continuous probability values of the ensemble SDMs were transferred into binary presence-absence predictions by applying species-specific mean cut-off values across the individual SDMs, as obtained from the biomod2 results as thresholds (**Table 1**, column 3). Species that showed suitability below this threshold at a given site were considered unsuitable and systematically assigned to a zero value. When calculating the climatic suitability for the native oak species, SDM data from the two individual species *Q. petraea* and *Q. robur* were used and aggregated to *Quercus* spp. in order to match to the data of species compositions and growth performance of the Austrian forest inventory which does not differentiate between these species. Consequently, for the future species projections at each centroid, those oak species was selected that had a higher suitability based on the established threshold.

Tree species that are occurring today according to the forest inventory but were found to be unsuitable for the RCP 4.5 or 8.5 scenario at a given centroid, were considered to be unable to regenerate and to become locally extinct in the long term. The vacated space at the respective centroid was either filled with other suitable tree species or left unstocked if none of the 15 studied native or non-native species could be identified as suitable. The potential planting of tree species at vacant sites was simulated according to five different species change scenarios (**Table 2**).

**Table 2:** The five modelled species change scenarios.

Tree species change scenario	Use of non-native species	Species selection criteria
‘No adaptation’	–	No adaptations take place. Failing tree species can only be replaced with other species already occurring at a given plot.
‘Native-MSS’	No	Select species with the highest climatic suitability (quantified relative to its respective cute-off threshold). MSS, most suitable species.
‘Combi-MSS’	Yes	Select species with the highest climatic suitability (quantified relative to its respective cut-off threshold). MSS, most suitable species.
‘Native-CC/BB’	No	Replace conifers preferable by conifers and broadleaved trees preferably with broadleaved trees (=CC/BB). In both cases, species with the highest suitability above the cut-off threshold were selected. If no conifer, respectively broadleaved species was suitable, species with the highest climatic suitability were selected irrespective of being conifer or broadleaved.
‘Combi-CC/BB’	Yes	Replace conifers preferable by conifers and broadleaved trees preferably with broadleaved trees (=CC/BB). In both cases, species with the highest suitability above the cutt-off threshold were selected. If no conifer, respectively broadleaved species was suitable, species with the highest climatic suitability were selected irrespective of being conifer or broadleaved.

The scenarios were modelled for the timespan 2081–2100 (RCP 4.5 and 8.5).

### 2.3 Tree species change scenario

To calculate the potential future tree species composition and the resulting contributions to the ecosystem services, we considered several tree species change scenarios (**Table 2**). These scenarios are based upon the modelled probability of occurrence of the tested native and non-native species and their share within the forest inventory. We assume that tree species that were modelled to be not suitable (respectively absent) under future climate will get locally extinct throughout the course of the next decades. Depending on the share of this failing tree species in current forests, this may result in a loss of the considered ecosystem services.

The first scenario, referred to as ‘no adaptation’, represents the zero variant where no active adaptation of the species composition to climate change would occur. Thus, failing species could be replaced only by species which already occur today at the respective inventory centroid given that these species were found to be suitable. Otherwise, the centroid will be left unstocked. All other scenarios presume active adaptation by reforesting failing tree species either by suitable native tree species or by suitable NNT. In total four active adaptation scenarios were defined, which differ in terms of its consideration of NNT and in terms of replacing failing species either with the most suitable tree species or by replacing conifers preferably with suitable conifers and broadleaved trees preferably with suitable broadleaved trees. Centroids for which no climatically suitable species could be identified among the available tree species spectrum, would be left unstocked.

### 2.4 Tree species composition

To depict the current composition of tree species, we calculated the frequencies of all native and non-native species across the inventory centroids. It is important to note that the present analysis focuses on seven stand-forming and most notable native species that cover in total around 84% of the stocked and managed forest area and present around 90% of the growing stocks in Austrian forests. Other native species are mostly minor, rare and scattered distributed species (i.e. *Acer spec*., *Sorbus spec*.) or face endangering diseases (e.g. *Fraxinus spec, Ulmus spec*.). Therefore, these species are not assumed to fully replace potentially climatically unsuitable stand-forming species and their contributions to ecosystem services. Also, this minor species lack sufficient data on potential distribution and growth potential and were not considered in the present analysis. To quantify the tree species diversity at a given inventory centroid, the tree species richness (number of tree species per plot) was calculated.

### 2.5 Forest productivity

To estimate the potential value of future species composition to provisioning ecosystem services, we used the tree species’ annual increment as productivity indicator (**Table 1**). The mean annual increment has been obtained for the different species either on basis of the Austrian forest inventory for the native species or by mean values calculated from published studies of NNT (**Table 1**). To obtain a dimensionless contrast among species, the annual volume increment has been standardized across the mean increment of all species. Although the actual productivity at a given centroid is strongly affected by local site conditions, tree age, management etc., our basic analysis assumed the annual increment to be equal across all centroids and the course of ongoing environmental changes.

The standardized increment value of the tree species apparent on the centroid were averaged and the centroids are categorized into three groups: high production (>0.78), moderate production (0.63–0.78), and low production (< 0.63). These categories were defined by the two native tree species with the highest increment (*Abies alba* and *Picea abies*) and the two with the lowest increment values (*Pinus sylvestris* and *Larix decidua*) (**Table 1**).

### 2.6 Protection against natural hazards

#### 2.6.1 Avalanche control

Avalanche risk assessment commonly considers stand proximity, tree age, forest structure, altitude, and slope characteristics (Margreth, 2004). In this study, avalanche risk was specifically calculated for centroids situated at altitudes above 800 m with slopes ranging from 25° to 60° (Schneebeli and Bebi, 2004). The risk evaluation was based on the proportion of wintergreen trees (*P. abies, A. alba*, and *P. sylvestris*) within each centroid, with a higher share indicating a lower avalanche risk due to increased interception. Notably, wintergreen stands can have larger openings than deciduous forests before avalanches occur, rendering them advantageous, particularly in warmer temperatures (Schneebeli and Bebi, 2004; Teich et al., 2012).

Another important factor to consider is the stand density index. Since only data on stand volume was available, we set a threshold value of 100 m³ over bark/ha. We assume that an area is not fully stocked when the value of stand volume is less than 100 m³ over bark/ha (**Table 3**). When the proportion of wintergreen species was equal or above 70% the avalanche risk was categorized as “low” and when the proportion was below 40% the risk was “high” (BASCH, 2021). If species with high volume in the present inventory were found to be unsuitable in the future, the total stand volume was reduced by the respective share and the changes over time estimated as a percentage of the current proportion.

**Table 3:** Criteria for assigning avalanche and rockfall hazard categories.

Risk	Avalanche	Rockfall
**low**	≥ 70% wintergreen species	Volume ≥ 360 m³
**moderate**	41 – 69% wintergreen species	Volume 181–359 m³
**high**	≤ 40% wintergreen species or Volume ≤ 100 m³	Volume ≤ 180 m³

#### 2.6.2 Rockfall control

Crucial stand attributes for effective rockfall protection include tree diameters and stem numbers (Dorren et al., 2005). Recognizing that the forest’s ability to intercept falling rocks correlates with stand density, we approximated density using stock volume. We chose this approach because only volume data was available to us. Centroid stock volumes ranged from 0 to 1700 m³ over bark/ha. Centroid with volumes surpassing the mean of 360 m³ over bark/ha were classified as low risk, while those with volumes below 180 m³ over bark/ha were considered high risk (**Table 3**). This categorization is based broadly on recommendations for action by forest protection experts (BASCH, 2021). The rockfall risk assessment was exclusively conducted for centroids located in hazard zones, where rockfall incidents had occurred in recent years (ROCKtheALPS, 2019), encompassing both release and deposit areas. Only centroids overlapping these layers were considered. Changes over time were calculated and presented as a percentage of the current volume.

## 3 Results

### 3.1 Tree species composition

**Figure 2:**
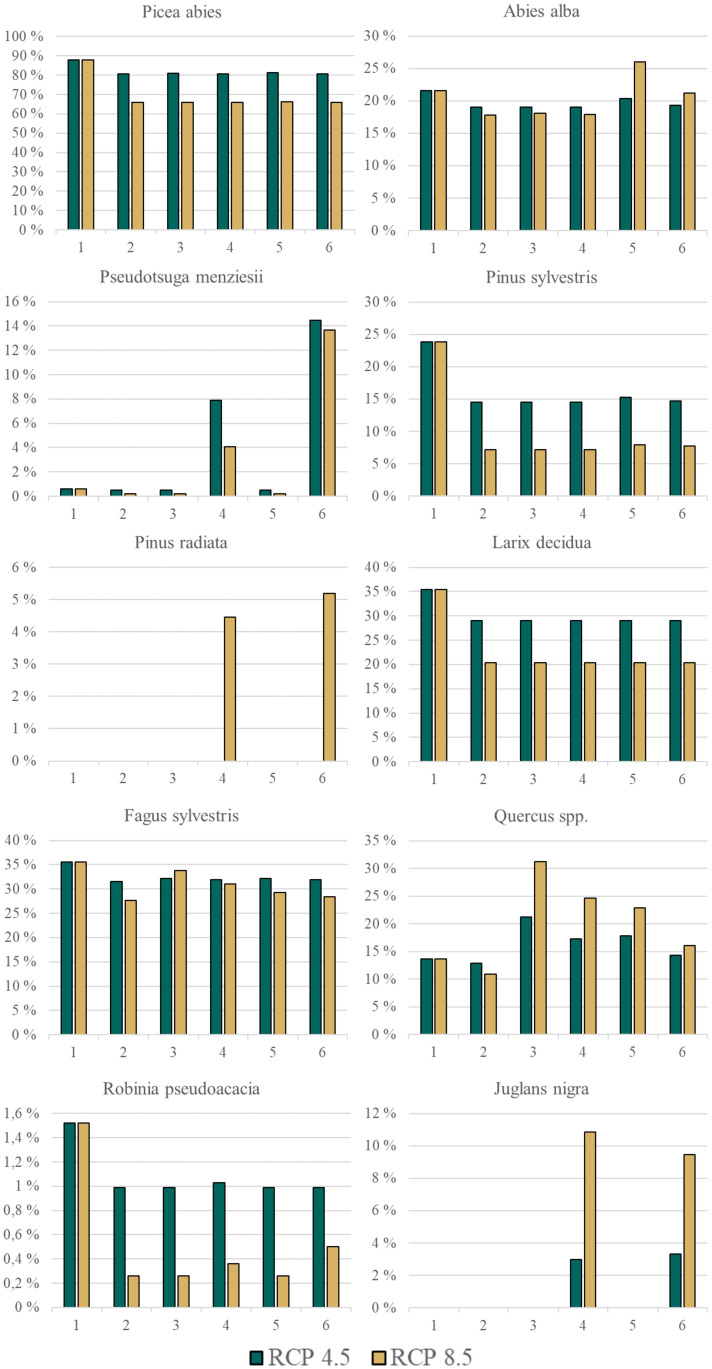
Frequency of occurrence of a species in Austrian forests (as estimated for the Austrian forest inventory clusters) for 10 of the 15 analysed tree species (also see **Table 1**) in Austria. Number code for species change scenario: 1 = current, 2 = no-adaptation, 3 = Native-MSS, 4 = Combi-MMS, 5 = Native CC/BB and 6 = Combi CC/BB. Please be aware of the different y-axes for each species. ‘Current’ displays the current situation observed in the inventory, the five species change scenarios display the situation in 2081 under future climate (RCP 4.5 and 8.5). Five species are not represented as their frequency is always below 0.5% (*Thuja plicata, Abies grandis, Fraxinus pennsylvanica, Pinus contorta, Quercus rubra*); (Native): only native species are used; (Combi): Native and non-native species are used; (MSS): a tree species that fails is replaced by the most climatically suitable species, either coniferous or broadleaved; (CC/BB): coniferous species are primarily replaced by coniferous species and broadleaved species are primarily replaced by broadleaved species.

*P. abies* is the most common species at the Austrian forest inventory now and will remain so in the future, regardless of the tree species change scenario (**Figure 2**). The frequency of *L. decidua* and *P. sylvestris* on forest inventory plots is expected to decrease in the future compared to the present. The distribution area of native oaks (*Q. petraea & Q. robur*) in Austria is expected to expand strongly in the future, thus increasing their frequency in the total forest area. *J. nigra* and *P. menziesii* are the most frequent NNT. *R. pseudoacacia* is modelled to occur only on 0.26 - 0.5% of the forest area in the future. The NNT *A. grandis*, *F. pennsylvanica*, *P. contorta*, and *Q. rubra* only show low share in present forests and are also expected to occur only rarely in the future.

**Figure 3:**
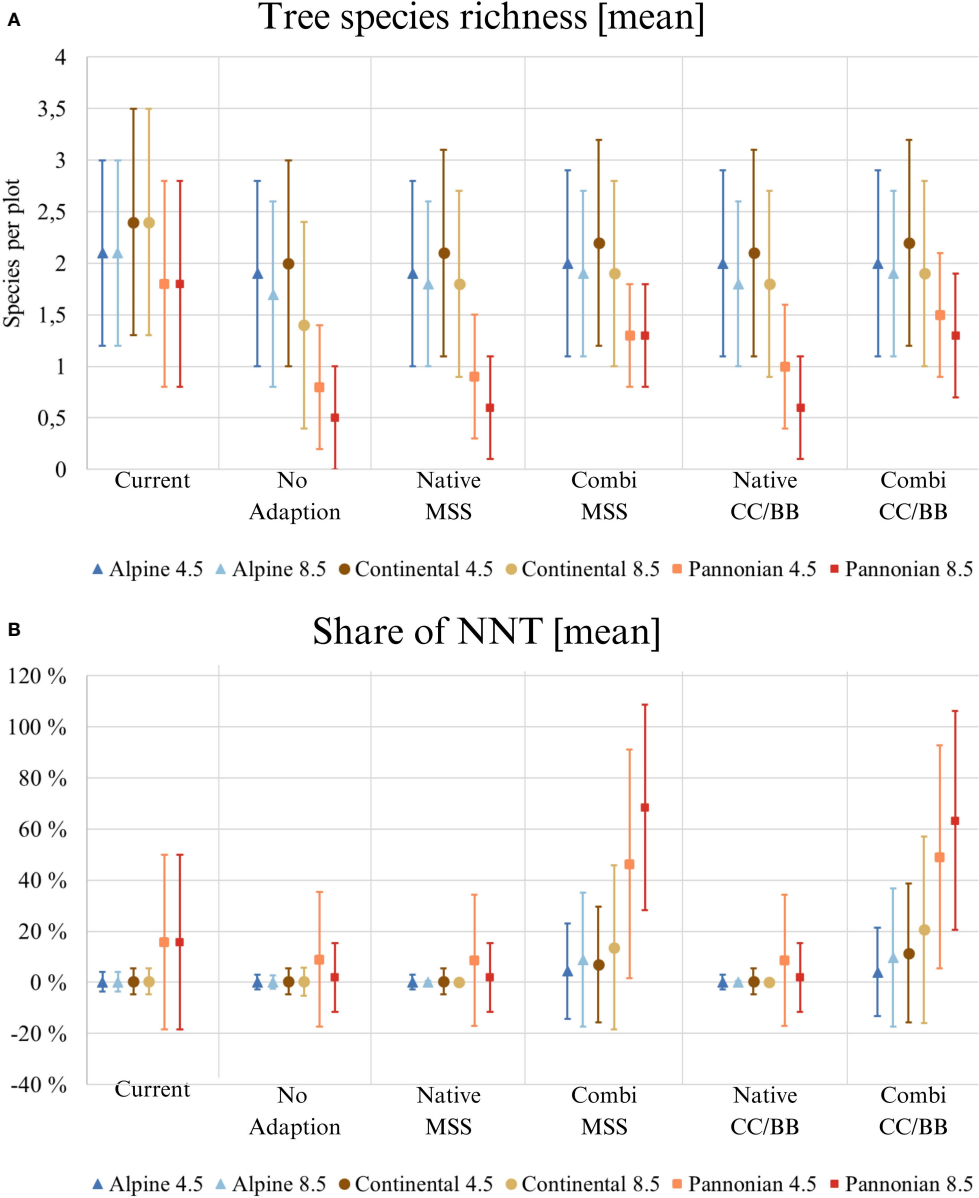
Results of tree species composition modelling across Austria. Applies to all three illustrations: ‘Current’ displays the current situation with the present climate, the five species change scenarios display the situation in 2081 under future climate (RCP 4.5 and 8.5). The mean value, along with its standard deviation, is always given. (Native): only native species are used; (Combi): Native and non-native species are used; (MSS): a tree species that fails is replaced by the most climatically suitable species, either coniferous or broadleaved; (CC/BB): coniferous species are primarily replaced by coniferous species and broadleaved species are primarily replaced by broadleaved species. Please be aware of the different y-axes for each category. **(A)** Tree species richness shows the average number of tree species (from **Table 1**) per study site; **(B)** Share of NNT [%] (NNT, non-native tree).

The predicted composition of tree species varies considerably according to the specific species change scenario and climate scenario in question (**Figure 2**). For instance, *Pinus radiata* is present in the CC/BB scenarios in RCP 8.5, but not in any other combination of species change and climate scenario (**Figure 2**). In contrast, for other tree species such as *Picea abies* and *Pinus sylvestris*, the decisive factor is the climate scenario. The highest tree species richness can be observed under current conditions, indicating that climate change both with and without active adaptation measures will result in a substantial decline of tree species richness (**Figure 3A**). It is also clear that the more extreme climate scenario RCP 8.5 has a more negative impact on tree species richness than the scenario RCP 4.5. Differences in the impact on different environmental zones are also clearly visible. The negative consequences for the Pannonian are the most prominent, while in contrast, the Continental Zone and the Alpine South exhibit a markedly reduced range of variation in the differences between the individual species change scenarios.

The mean share of NNT in Austrian forests is around 1% at present in de Alpine South and the continental zone, while in the Pannonian it nearly reaches 20% (**Figure 3B**). Without active adaptation or if adaptation is excluding NNT, the mean share of NNT may drop down in all three zones. As expected, including NNT in forest adaptation increases their mean share. The proportion of NNT would clearly be highest in Pannonian and could be over 60% under RCP 8.5.

### 3.2 Forest productivity

**Figure 4:**
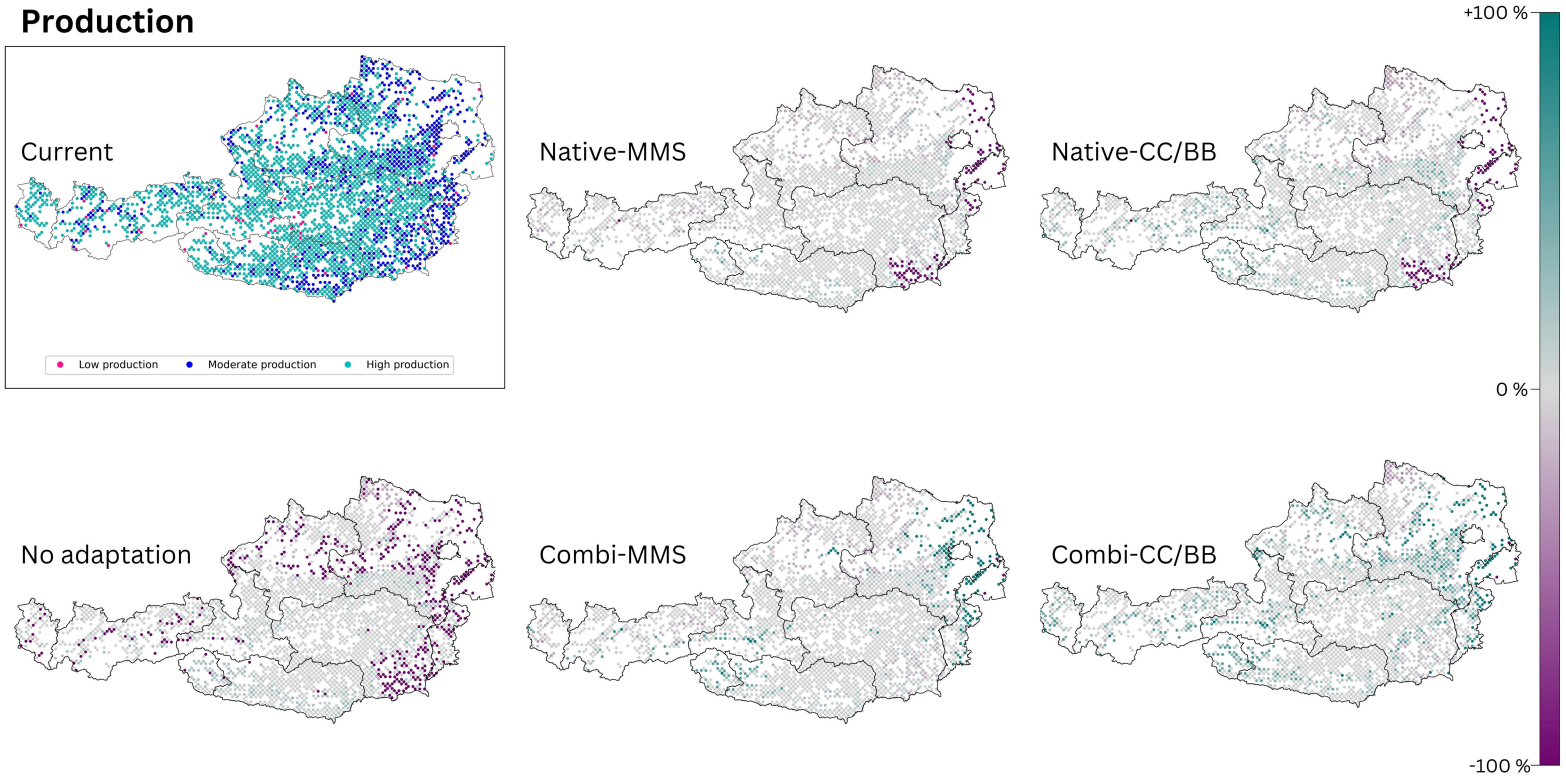
The production output of today’s climate is compared with those of the five species change scenarios for future climate (2081–2100, RCP 8.5). The section titled ‘current’ displays the present status of the analysed ecosystem service. The effects of the 5 species change scenarios are also shown. Purple dots indicate a negative change compared to the current state, and turquoise dots indicate a positive change. (Native): only native species are used; (Combi): Native and non-native species are used; (MSS): a tree species that fails is replaced by the most climatically suitable species, either coniferous or broadleaved; (CC/BB): coniferous species are primarily replaced by coniferous species and broadleaved species are primarily replaced by broadleaved species.

**Figure 5:**
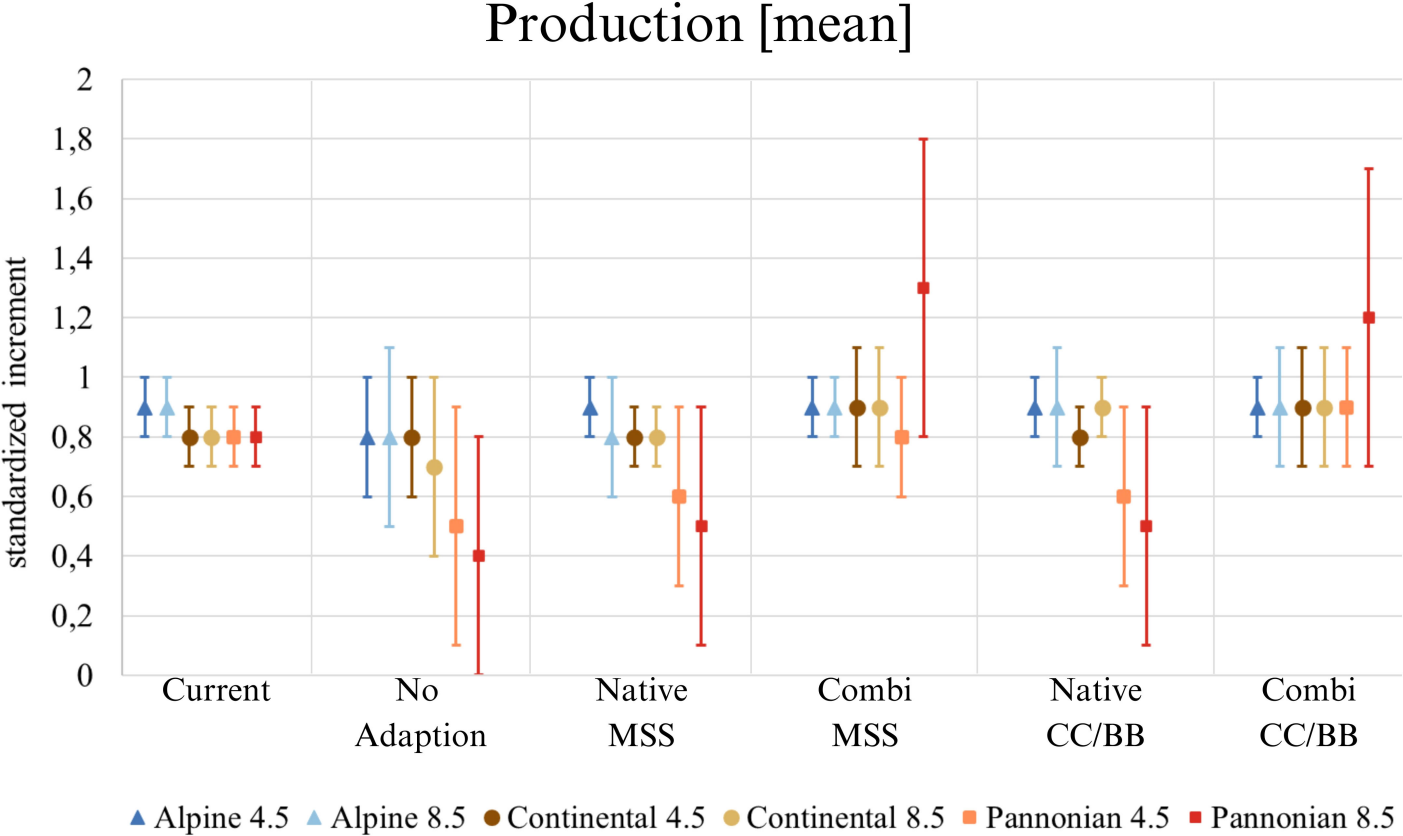
Mean production value (increment per plot) across Austria. ‘Current’ displays the situation with the present species composition, the five species change scenarios display the situation in 2081 under future climate (RCP 4.5 and 8.5). The mean value, along with its standard deviation, is always given. (Native): only native species are used; (Combi): Native and non-native species are used; (MSS): a tree species that fails is replaced by the most climatically suitable species, either coniferous or broadleaved; (CC/BB): coniferous species are primarily replaced by coniferous species and broadleaved species are primarily replaced by broadleaved species.

Our results indicate that timber production would decrease without adaptation to climate change (**Figure 4**). Areas that would be affected by a loss of production are located mainly in at the Pannonian zone and the Continental zone, but also within inner alpine valleys in western Austria, which are climatically characterized by dry continental climate (**Figure 4**). If only native tree species are used for adaptation, the mean increment in the Alpine south and the Continental zone would remain similar as it is today or only change slightly, while it would clearly decrease in the Pannonian zone (**Figure 5**). The map indicates that a limited number of study sites may exhibit a slight increase in timber production when native conifers are replaced with other native conifers and native broadleaves with native broadleaves (Native-CC/BB). However, the overall number of moderately and highly productive study sites appears to decline (**Table 4**). If NNT are included as adaptation option, the mean production value of the Continental and Pannonian zone and the total number of high productive study sites for Austria would be higher than it is today (**Table 4**). This would be especially beneficial in areas that already have low productivity today.

**Table 4:** Number of plots per species change scenario for [A] production (N=3024), [B] avalanche control (N=885) and [C] rockfall control (N=524).

Production [A]	Low production		Moderate production		High production	
	RCP 4.5	RCP 8.5	RCP 4.5	RCP 8.5	RCP 4.5	RCP 8.5
Current	58	58	829	829	2137	2137
No adaptation	196	512	721	586	2107	1926
Native-MSS	69	133	841	961	2114	1930
Combi-MSS	86	29	808	908	2130	2087
Native-CC/BB	38	173	719	809	2267	2042
Combi-CC/BB	42	65	580	650	2402	2309
Avalanche [B]	High risk		Moderate risk		Low risk	
	RCP 4.5	RCP 8.5	RCP 4.5	RCP 8.5	RCP 4.5	RCP 8.5
Current	141	141	146	146	598	598
No adaptation	140	179	137	117	608	589
Native-MSS	128	186	137	149	620	550
Combi-MSS	128	178	136	138	621	569
Native-CC/BB	128	124	136	117	621	644
Combi-CC/BB	128	124	136	117	621	644
Rockfall [C]	High risk		Moderate risk		Low risk	
	RCP 4.5	RCP 8.5	RCP 4.5	RCP 8.5	RCP 4.5	RCP 8.5
Current	104	104	185	185	235	235
No adaptation	117	152	181	181	226	191
Native-MSS	104	104	185	185	235	235
Combi-MSS	122	104	200	185	172	235
Native-CC/BB	104	104	185	185	235	235
Combi-CC/BB	122	104	200	185	172	235

‘Current’ displays the current situation with the present climate, the five species change scenarios display the situation in 2081 under future climate (RCP 4.5 and 8.5). (Native): only native species are used; (Combi): Native and non-native species are used; (MSS): a tree species that fails is replaced by the most climatically suitable species, either coniferous or broadleaved; (CC/BB): coniferous species are primarily replaced by coniferous species and broadleaved species are primarily replaced by broadleaved species.

For reasons of space, only the maps for RCP 8.5 are presented here (**Figure 4**). The map for RCP 4.5 (see **Supplementary Figure S5**) exhibit a comparable pattern to that observed in RCP 8.5, albeit to a lesser extent.

### 3.3 Protection against natural hazards

**Figure 6:**
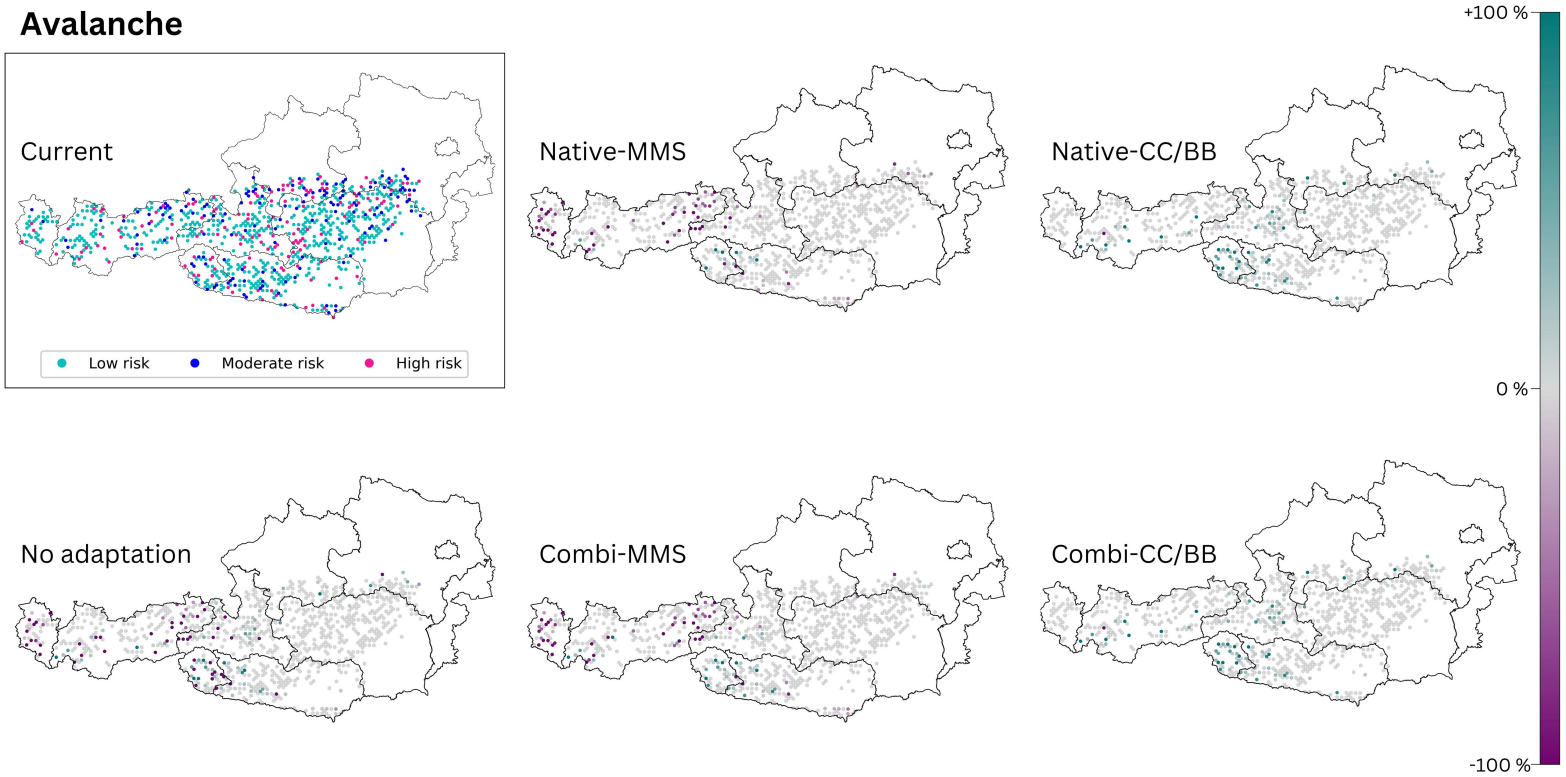
The avalanche risk of today’s climate is compared with those of the five species change scenarios for future climate (2081–2100, RCP 8.5). The section titled ‘current’ displays the present status of the analysed ecosystem service. The effects of the 5 species change scenarios are also shown. Purple dots indicate a negative change compared to the current state, and turquoise dots indicate a positive change. (Native): only native species are used; (Combi): Native and non-native species are used; (MSS): a tree species that fails is replaced by the most climatically suitable species, either coniferous or broadleaved; (CC/BB): coniferous species are primarily replaced by coniferous species and broadleaved species are primarily replaced by broadleaved species. The number of plots was limited to those where avalanches are possible (see methods section). This restriction resulted in blank areas on the map.

**Figure 7:**
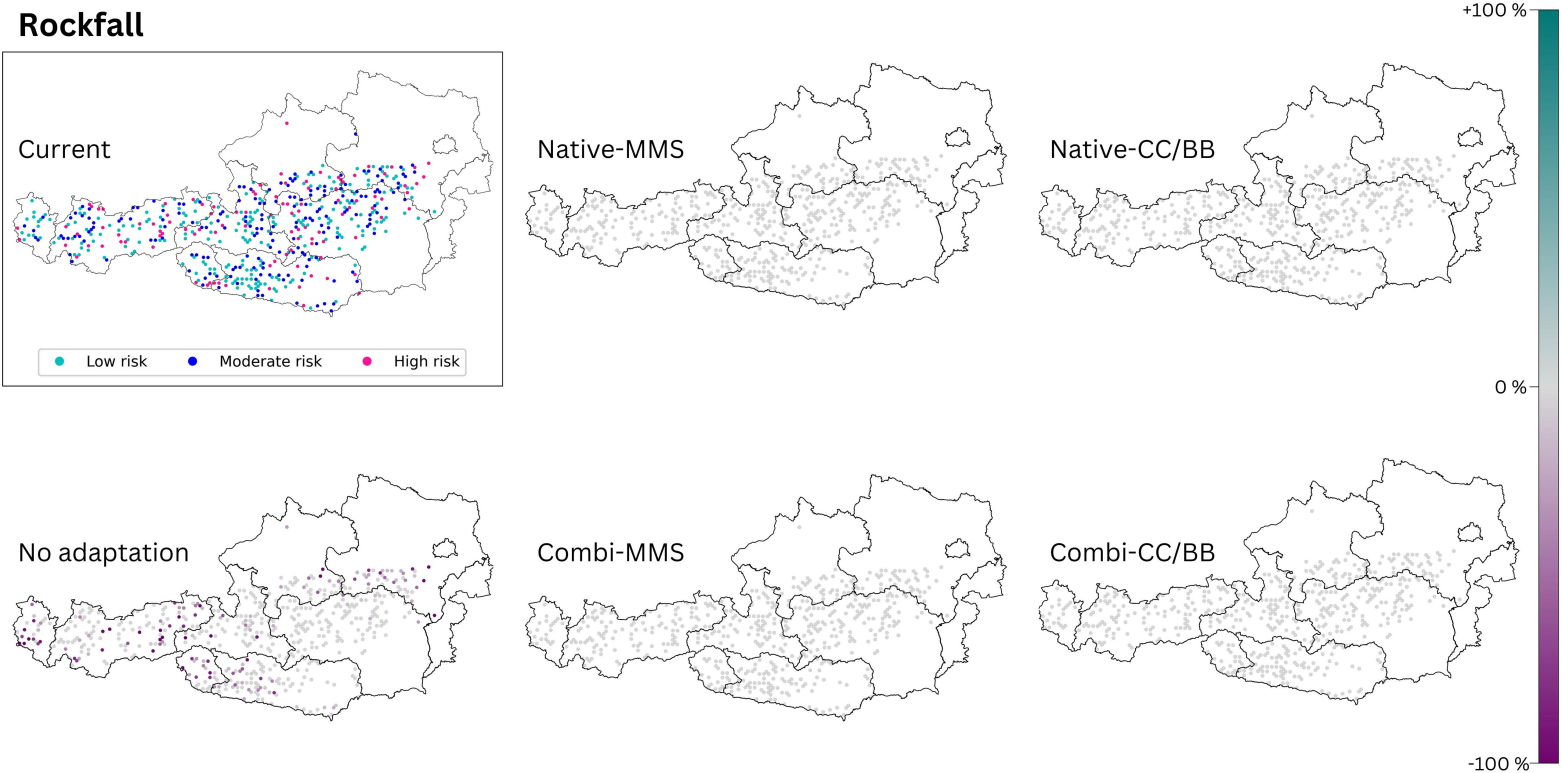
The rockfall risk of today’s climate is compared with those of the five species change scenarios for future climate (2081–2100, RCP 8.5). The section titled ‘current’ displays the present status of the analysed ecosystem service. The effects of the 5 species change scenarios are also shown. Purple dots indicate a negative change compared to the current state, and turquoise dots indicate a positive change. (Native): only native species are used; (Combi): Native and non-native species are used; (MSS): a tree species that fails is replaced by the most climatically suitable species, either coniferous or broadleaved; (CC/BB): coniferous species are primarily replaced by coniferous species and broadleaved species are primarily replaced by broadleaved species. The number of plots was restricted to those where rockfall has already occurred (see methods), resulting in blank areas on the map.

The criteria for the selection of study sites at which the avalanche and rockfall control is analyzed (see methods section) result in the study sites being limited to the Alpine South and the Continental Zone. For reasons of space, only the maps for RCP 8.5 are presented here (**Figures 6**, **7**). The maps for RCP 4.5 (**Supplementary Figures S6, 7**) exhibit a comparable pattern to that observed in RCP 8.5, albeit to a lesser extent.

#### 3.3.1 Avalanche control

‘No adaptation’ yields results similar to those of Native-MSS and Combi-MMS (**Figure 6**). In all three scenarios a deterioration of the protective function in the western part of Austria, can be expected. If coniferous species are mainly replaced by coniferous species and broadleaved species are mainly replaced by broadleaved species (CC/BB) an improvement of the current protective function is anticipated (**Table 4**). It does not seem to make a difference in outcomes whether in the CC/BB scenario only native species or a combination of native and NNT are used for adaptation.

#### 3.3.2 Rockfall control

Only when no adaptation takes place in the Austrian forest, a deterioration of the current situation of rockfall protection can be observed (see **Table 4**). If one of the other four species change scenarios is applied, there is no difference to the current status of this protective function (**Figure 7**).

## 4 Discussion

In Austrian forests, seven major tree species cover around 84% of the forest area and provide manifold ecosystem services such as timber production, carbon sequestration and protection of infrastructure and settlements from natural hazards such as rockfall and avalanches. Our analysis indicated that in climate change - without adapting the tree species composition – a wide-ranging loss of ecosystem services and a decrease in climatic suitability of tree species, which could lead to a decrease in tree species richness can be expected. Promoting forests’ adaptation with native species will be able to maintain the forests protective ecosystem services in mountainous forests but will result in declines of productivity and carbon sequestration. However, considering also the use of NNT for adaptation on up to 18% (average value for Austria) of the forests area could help sustain forest productivity (RCP 8.5).

NNT currently play a subordinate role in the Alpine south and the Continental zone, as they only representing one percent of the forest area there. In the Pannonian zone, on the other hand, the proportion of NNT is currently already 15.8% and is therefore significantly higher. In view of the effects of climate change, the importance of NNT in Austrian forestry could increase drastically not only in the Pannonian, but also in the two other zones. Our analyses also show that NNT are particularly important for productivity in commercial forests. In protective forests, timber yield is only of secondary importance. The primary function there is the protection of people and infrastructure from natural hazards. The analyses of the protective functions of avalanche and rockfall suggests that these protection forests can maintain their primary functions by promoting the growth of climatically suitable native species. According to our results using NNT does not provide superior protection compared to using native species.

### 4.1 Climate change impacts on tree species composition

Due to the topographical diversity in Austria, the forest types vary naturally greatly among the Alpine south, the Continental and Pannonian zone. Our findings indicate that the dry lowland in Austria’s east, which is part of the Pannonian zone, will particularly face the consequences of the changing climate. For many decades, coniferous tree species were planted for forest management there. These include *Pinus sylvestris*, *Picea abies*, *Larix decidua* and *Abies alba*. Our predictions indicate that these conifers, along with *Fagus sylvatica*, will no longer thrive in the future due to unsuitable climatic conditions in the Pannonian zone. As a result, the tree diversity and timber yield of the forests in the region will be drastically reduced. If native tree species are unable to migrate to higher altitudes, as is the case in the lowlands of Austria, they are likely to face extinction in the region (Jandl et al., 2019). One way to counteract the loss of timber production in lowlands (Continental and Pannonian zone) with future climate could be the use of NNT (Brang et al., 2016) and our results show potential benefits of such an approach. Especially, lowland areas with low rainfall, like the Pannonian zone, could benefit of a combination of native species and NNT (**Figure 3**).

As shown in **Figure 2**, the good climatic suitability of native oaks is evident under future climatic conditions. The two native species, in particular *Q. petraea*, are species of the colline and montane stages (Aas, 2014a, 2014b). This makes them the ideal native species for the management of lowland forests. The non-native red oak (*Q. rubra)* is not a suitable replacement species in these forests, as it is less suited to the climate and could also cause conservation problems (Dreßel and Jäger, 2002; Vor et al., 2015). Therefore, the native oaks should clearly be favored over the non-native *Q. rubra*. To achieve a balanced mix of tree species, also other tree species should be cultivated next to native oaks in lowland forests. One of the most popular tree species for mixing with oaks is the native species hornbeam (*Carpinus betulus*) (Ruhm et al., 2021; Schönauer and Schüler, 2021). However, due to the anticipated reduction in the suitable range for hornbeam (Varol et al., 2022), this combination may not be possible in the future. According to our results, tree species such as *Pinus sylvestris* or *P. radiata* could be suitable mixed tree species for oak forests.

According to the results, a decrease in the proportion of Black locust (*Robinia pseudoacacia*) can be expected in both climatic scenarios. This decrease may be caused by the models used here, as the study sites only represent forest area and not open land. While climatic suitability is a main driver of a species’ distribution other factors such as its competitive strength (e.g. rapid growth and change in soil chemistry due to nitrogen deposition) are not considered in our calculations. Black locust is highly competitive in open land (Huntley, 1990) and considered invasive in many European countries, including Austria (Essl and Rabitsch, 2002). Due to its additional good adaptation to drought, black locust is expected to thrive in Austria’s changing climate (Mantovani et al., 2014; Vítková et al., 2017; Nicolescu et al., 2020). Contrary to our results, an increase in the occurrence of *R. pseudoacacia* in Austria can therefore be expected in the future, especially in open land (Kleinbauer et al., 2010; Li et al., 2014). *R. pseudoacacia* is known for its tendency to colonize open land (Huntley, 1990). The data only refers to Austrian forests and do not reflect this occurrence. Therefore, it can be assumed that the black locust is more widespread in Austria today than the data shows. Nevertheless, the reliability of the National forest inventory data is remarkable for tree species of low abundance. The data show a meaningful correlation with actual occurrences, underlining the credibility and applicability of the method even for less common tree species.

Besides their potential to provide ecosystem services in commercial forests, NNT can pose several risks. NNT can become invasive, spreading from planting sites into areas of high ecological value and sometimes causing irreversible damage to biodiversity and related ecosystem services (Richardson et al., 2000; Richardson and Rejmánek, 2011; Castro-Díez et al., 2019). The recommended use of site-specific risk assessments helps to detect and evaluate the risk response and intensity under certain site conditions (Bindewald et al., 2021; Lapin et al., 2023).

*Douglas fir (P. menziesii)* is one of the most used NNT in Europe. It has no known negative impacts on Central European forest communities except on two (*Luzulo-Quercetum petraeae* HILITZER 1932 and *Deschampsia flexuosa-Acer pseudoplatanus* - Association) (Bauhus et al., 2017; Bindewald and Michiels, 2016). Western Redcedar (*T. plicata*), which can grow in a range of habitats (Antos et al., 2016), has been categorized as invasive in the UK (Richardson and Rejmánek, 2004) and potentially invasive in Belgium (Fanal et al., 2021). Unlike the Douglas fir and the Western Redcedar, the grand fir (*A. grandis*) does not appear to have the potential to invade any habitats in Central Europe (Spellmann et al., 2015). The Monterey pine (*P. radiata*), one of the most frequently cultivated species of pine (McDonald and Laacke, 1990), is already considered invasive in some countries (Higgins and Richardson, 1998; Lindenmayer and McCarthy, 2001; Williams and Wardle, 2009) and the lodgepole pine (*P. contorta*), is widely regarded as the most invasive *Pinus* species in the southern hemisphere (Davis et al., 2019). Due to its ability to allelopathize (Rietveld, 1983), black walnut (*J. nigra*) can have a negative effect on the native species composition of the understory (Nicolescu et al., 2020). And the last of the nine non-native species, the green ash (*F. pennsylvanica*), is so ecologically adaptable that it can take advantage of almost any opportunity to invade, with floodplain habitats being particularly favorable (Drescher and Prots, 2016).

In the case of *T. plicata, P. contorta, Q. rubra* and *F. pennsylvanica*, both the results of the modelling carried out here and the potential invasive risks speak for themselves. These species are not suitable as future tree species in the face of climate change for either the Alpine south, the Continental or the Pannonian zone. Due to its high risk of invasion, *R. pseudoacacia* should also not be encouraged in forestry. Forestry utilization can be considered for the NNT species *P. menziesii, A. grandis, P. radiata* and *J. nigra*. However, it is important to take a number of management measures into account when cultivating NNT for forestry purposes (Marinšek et al., 2022). For instance, NNT should only be cultivated in a mixture with native species.

### 4.2 Climate change impacts on forest productivity

It is becoming clear that forest management will need to adapt to climate change in order to counteract potentially declining timber production in the future. The continued use of native species such as Norway spruce (*P. abies*) can maintain production levels in Alpine South of Austria at least comparable to current levels. However, the situation in the Pannonian zone and partly in the Continental zone is different (**Figure 4**). Due to climate change and resulting calamities (e.g. bark beetle outbreaks or storm events), there is a growing need to adapt species selection in forestry. The current dominance and the expected decrease of Norway spruce - known for its high yield – is thus one driver of lower future timber production (Hanewinkel et al., 2010). Especially, secondary pure stands of Norway spruce planted in lowlands beyond its native habitat (Spiecker, 2004).

Many forest managers are now using a balanced mixture of Norway spruce and other (broadleaved) tree species when replanting secondary Norway spruce forests (Russ, 2019) (Hanewinkel et al., 2010). The use of both native and NNT can sustain production levels. In some cases, this combined approach can even lead to increased production compared to current levels. Specifically, if coniferous tree species are preferred as a replacement for declining conifers (CC/BB), a substantial increase in production (increment) compared to today can be expected in the Continental zone. The reason for this higher productivity can be explained by the change in species composition. Our results indicate that Norway spruce trees at secondary sites, where they might grow suboptimal, are being replaced by more productive and better suited species (*A. alba, P. radiata* und *P. menziesii*). Anyhow, an extended plantation non-native trees such as *P. menziesii* or *P. radiata* will also require adaptations in the Austrian timber industries as they provide different wood qualities and assortments and might therefore be suitable for other timber applications (Huber et al., 2023). However, reforestation action in the coming decades will only deliver sawnwood by the end of the century, which gives sufficient times for wood industries to update its technologies as innovations may take 25–30 years to reach the market (Teischinger and Müller, 2010). Besides wood qualities, the consideration of forest growth and productivity is of utmost importance, as the value chain from the European forestry industry and wood sector totals around 1.1 trillion euros (EU 27, Norway, Switzerland, United Kingdom). In total, the industry thus secures around 17.5 million jobs across Europe (Econmove and Economica, 2023). Therefore, the forest and its resources, specifically wood, play a significant role in the domestic economy.

Another important ecosystem service related to forest productivity is the forest carbon sink, i.e. the sequestration of carbon from the atmosphere, which was recently found to be strongly reduced by the loss and future lack of native tree species (Wessely et al., 2024). Although, we have not quantified the forest carbon sink explicitly, the improved productivity of mixtures with native and non-native conifers, would also reveal the highest carbon sink and thus help to improve or continue the mitigation of climate change (Lorenz and Lal, 2010).

### 4.3 Climate change impacts on protective forest

The protective function is of great importance throughout the Alps. This importance is illustrated here by the example of Austria. In Austria, almost every fourth citizen benefits from the function of protective forests (BML, 2023). In total 1.6 million hectares, or 42% of Austria’s forest area, are classified as protective forests (BML, 2023). Over 25% of these forests are directly protecting infrastructure and settlements against geohazards such as avalanches and rockfalls (Berger et al., 2013). Our results show that the selection of appropriate tree species depends on the primary protective function of the forest and varies by objective, i.e. whether protection from avalanches or rockfall are more likely to occur. It is also worth mentioning that that mountain forests are widely recognized as the most effective, cost-efficient, and aesthetically pleasing form for protecting against natural hazards (European Observatory of Mountain Forests, 2000).

#### 4.3.1 Avalanche control

When a failing conifer species is replaced with a more climate-favorable conifer species during climate adaptation, the protective function improves compared to the present (**Figure 6**). We show that choosing an evergreen or deciduous species is more important for avalanche protection than the choice of the actual tree species. While Schneebeli and Bebi (2004) have demonstrated that the stability of snow cover in evergreen forests and deciduous forests is similar, we found that the shift of the forest cover towards evergreen species enhanced the protection situation. A cross-national project between Austria and Germany also recommends increasing the proportion of evergreen species in forests when it comes to preventing avalanches (BASCH, 2021). Nevertheless, our result is clearly limited through this analytical approach. For example, the prediction without climate adaptation (‘no adaptation’) suggests an improvement in the protective function in some areas. However, this is misleading as the general degree of stocking decreases with this scenario. A lower stocking rate has a negative effect on avalanche protection because interception decreases and the open area on which avalanches can form increases (Schneebeli and Bebi, 2004; Teich et al., 2012). The analysis does not include the degree of forest cover. However, this information is essential for a more comprehensive assessment of the hazard potential. To gain a more comprehensive understanding of this protective function, future analysis should include additional parameters such as stocking degree, stand age, and structure.

#### 4.3.2 Rockfall control

None of the species change scenarios modelled led to a change in rockfall risk (**Figure 7**). The protective function is only reduced by the absence of climate change-adapted forest management due to the generally lower stocking level in this scenario. Results from other studies show that broadleaved tree species are better at absorbing the kinetic energy of rockfall than coniferous tree species (Dorren et al., 2005; Schmidt, 2005). Therefore, in our case, the MSS-scenarios should be favored as it has a higher frequency of broadleaved trees (**Figure 3**). Depending on the forest type, experts also recommend a minimum of 400–600 trees/ha with a diameter at breast height (DBH) > 12 cm for reliable protection against rockfall with a stone diameter of 30 cm, and a minimum of 300–400 trees/ha (DBH >24 cm) for a stone diameter of 60 cm (BASCH, 2021). The decisive factor in our modelling is not the degree of stocking per se, but the number of cubic meters of stock in the area, which was used due to data availability. As with avalanche protection, a more comprehensive understanding of this protective function will require additional parameters such as stocking density, stand age and structure to be included in future modelling.

### 4.4 Limitations

Modelling and visualizing future forest development in the face of climate change is crucial for forest managers and decision makers to develop adaptation strategies. However, it is important to interpret the modelling results objectively, as each approach is based on specific assumptions and development scenarios. The study was conducted based on a climate change scenario that assumes a temperature increase of up to 4.3°C by the end of the century (RCP 4.5 and 8.5). Any deviation from this temperature increase assumption could render different results. The greatest source of uncertainty in our study is therefore the uncertainty surrounding our future climate. We were unable to consider other RCP models in this study, as the SDMs we used are currently only available for RCP 4.5 and RCP 8.5.

Another limitation of our study is the use of tree species distribution models for the end of the century in conjunction with current forest stands. This assumes that the current forest stands will remain relatively constant over the next few decades. However, it is uncertain whether this assumption is correct and depends on various factors, such as the current age distribution of forests, current harvesting regimes, and national and European policy decisions. In recent decades, there has been a continuous increase in forest area and stock in Austria (SChadauer, 1996; BMNT, 2018). The first signs of stock and carbon saturation have only been observed in the last decade. Therefore, it is unclear whether assuming a constant forest stock for the next 50 years is realistic. To clarify this question, a modelling study based on empirical forest growth models that takes all of these variables into account would be necessary.

It is also important to note that our analysis does not consider local site characteristics such as exposition or soil type, as these were not part of the SDMs we used. Sustainable forest management adapted to local conditions could enable the survival of tree species at their climatic limits, even if they are currently classified as non-viable in our models (e.g. Norway spruce on north-facing slopes at lower elevations). Therefore, is it important to consider the potential benefits of adapting forest management practices to local conditions. As the introduction of NNT is not without controversy, we further recommend that a site-specific risk assessment should always be carried out before introducing them (e.g. Bindewald et al., 2021).

## 5 Conclusion

Currently, the proportion of non-native tree species in Austria is negligible. However, their importance could increase in the future. This study demonstrates that integrating NNT can particularly benefit lowland forests, increasing both production performance and tree species richness. Our results indicate that the use of NNT does not yield a superior or inferior impact on protective functions compared to utilizing native species exclusively. For a more detailed analysis of the future contribution of forests to the ecosystem services investigated (timber production, avalanche control and rockfall control), a more extensive analysis than the one presented here would be desirable, especially for the protective function. Despite this, our results make it clear that the use of NNT will increasingly be constrained to commercial forests.

This study highlights the importance of climate-adapted forest management in mitigating the negative impacts of climate change on tree species composition and ecosystem services. It underlines the urgency for local, regional and global forest management to adopt adaptive practices, otherwise forest productivity and tree species diversity may decline. Preserving and sustainably managing forests contributes to safeguarding crucial ecosystem services and actively mitigating rising temperatures (IPCC, 2019). To effectively tackle these challenges, it is essential to implement adaptation strategies and sustainable forest management practices. This involves safeguarding existing forests and strategically implementing afforestation measures to restore damaged or unstocked areas.

## Data availability statement

The datasets presented in this article are not readily available because the data includes the coordinates of the Austrian forest inventory, which are not available to the public. Requests to access the datasets should be directed to (julia.konic@bfw.gv.at).

## Author contributions

JK: Conceptualization, Methodology, Project administration, Validation, Visualization, Writing – original draft, Writing – review & editing. CH: Data curation, Methodology, Validation, Visualization, Writing – original draft. EH: Visualization, Writing – review & editing. DC: Investigation, Methodology, Writing – review & editing. KL: Conceptualization, Funding acquisition, Writing – review & editing. SS: Conceptualization, Funding acquisition, Methodology, Supervision, Writing – original draft, Writing – review & editing.
