# Supplementary material for: The potential of non-native tree species to provide major ecosystem services in Austrian forests
Source: Front Plant Sci. 2024 Jul 1;15:1402601. doi: 10.3389/fpls.2024.1402601 (PMC11246965; doi:10.3389/fpls.2024.1402601)
Supplement: Supplementary file 1 [file DataSheet_1.docx]

Table S 1: Overview of some ecological characteristics, invasive potential and cultivation in Central Europe of the nine non-native tree species included in this study

| Species | Ecological characteristics [1] | Invasive potential [2] | Cultivation in Central Europe [3] | Source |
| --- | --- | --- | --- | --- |
| *Abies grandis,*  *(Canada, USA)* | Shade tolerant, thrives in a variety of soils, rarely occurs in pure stands | Unlikely at present | frequently cultivated Particularly suitable as a mixed species and as a stabilising species on both wetter and drier sites | ([1] Foiles et al., 1990; [1, 3] Ruetz, 2014; [2] Spellmann et al., 2015) |
| *Fraxinus pennsylvanica,*  *(Canada, USA)* | Occurs as a pioneer and climax species, large ecological amplitude | Reported as (potential) invasive | cultivated in hardwood floodplain forests | ([1, 3] Schmiedel, 2007; [2] Drescher and Prots, 2016) |
| *Juglans nigra,*  *(Canada, USA)* | Large ecological amplitude, fast-growing, competitive, shade-intolerant species, allelopathy | Can affect native biodiversity | frequently cultivated in hardwood floodplain forests | ([1] Rietveld, 1983; [2] Nicolescu et al., 2020; [1, 3] Schaarschmidt, 2004) |
| *Pinus contorta,*  *(Canada, USA, Mexico)* | Pioneer species, tap root system, more competitive as *P sylvestris* | Reported as invasive | Widely used in Scandinavia, the UK and Ireland as an alternative for Scots pine and spruce | ([1] Engelmark et al., 2001; [2] Davis et al., 2019; [1, 3] Stephan, 2014) |
| *Pinus radiata,*  *(USA)* | Shade intolerant, shallow root system, fast growth | Reported as potential invasive (eg Australia, New Zealand and Chile) | World's most planted pine | ([1] Watson and O’loughlin, 1990; [2] Lindenmayer and McCarthy, 2001; Williams and Wardle, 2007; [1, 3] McDonald and Laacke, 1990) |
| *Pseudotsuga menziesii,*  *(Canada, USA), Mexico)* | Grows in a wide range of climatic conditions, heart fibrous root system, high tolerance to soil conditions | Invasiveness only known for specific habitats | Very common in a wide range of habitats | ([2] Bauhus et al., 2017; Bindewald and Michiels, 2016; [1, 3] Hermann, 2014) |
| *Quercus rubra,*  *(Canada, USA)* | Pioneer behaviour in extreme sites, tap root system, comparable environmental needs as native oaks | reported as potential invasive | Cultivated in Europe since 1691, well established | ([1] Marinšek et al., 2022; [1, 3] Brauer, 2015; [2] Dreßel and Jäger, 2002; Vor et al., 2015) |
| *Robinia pseudoacacia*  *(Canada, USA)* | Very competitive and can alter soil chemistry, shade intolerant | reported as invasive | One of the most cultivated trees worldwide, in Europe since the 16th century | ([1] Huntley, 1990; [2] Essl and Rabitsch, 2002; [1,3] Schütt, 2014; [3] Marinšek et al., 2022) |
| *Thuja plicata*  *(Canada, USA)* | Adapted to a wide range of habitats, shallow root system, prefers semi-shade | reported as (potential) invasive | one of the best performing non-native tree species in trial forest plantations in Central and Western Europe | ([1] Antos et al., 2016; [2] Fanal et al., 2021; Richardson and Rejmánek, 2004; [1, 3] Wickler, 2014) |

Table S2: Bioclimatic variables used to calibrate the SDMs obtained from WorldclimV2 (Fick and Hijmans, 2017)

| Acronym | Variable |
| --- | --- |
| BIO1 | Annual Mean Temperature |
| BIO2 | Mean Diurnal Range (Mean of monthly (max temp - min temp)) |
| BIO3 | Isothermality (BIO2/BIO7) (×100) |
| BIO4 | Temperature Seasonality (standard deviation ×100) |
| BIO5 | Max Temperature of Warmest Month |
| BIO6 | Min Temperature of Coldest Month |
| BIO7 | Temperature Annual Range (BIO5-BIO6) |
| BIO8 | Mean Temperature of Wettest Quarter |
| BIO9 | Mean Temperature of Driest Quarter |
| BIO10 | Mean Temperature of Warmest Quarter |
| BIO11 | Mean Temperature of Coldest Quarter |
| BIO12 | Annual Precipitation |
| BIO13 | Precipitation of Wettest Month |
| BIO14 | Precipitation of Driest Month |
| BIO15 | Precipitation Seasonality (Coefficient of Variation) |
| BIO16 | Precipitation of Wettest Quarter |
| BIO17 | Precipitation of Driest Quarter |
| BIO18 | Precipitation of Warmest Quarter |
| BIO19 | Precipitation of Coldest Quarter |

Table S3. Description of the SDMs used to develop the potential distribution of the NNTs according to the ODMAP protocol (Zurell et al., 2020).

| **ODMAP elements** | **Contents** |
| --- | --- |
| **OVERVIEW** | |
| Model objective | **SDM Objective:** forecast/transfer  **Target output:** probability of occurrence of target tree species |
| Taxon | *Nine Non-native tree species of Europe: (see Table S1)* |
| Location | Europe |
| Scale of analysis | **Spatial extent (Lon/ Lat):**  Longitude: -32.65000 °E -69.44167 °E  Latitude: 30.877982 °N -71.57893 °N  **Spatial resolution:** 30 arcsec  **Temporal resolution:** We modelled for historic climate (1961-90) and three future time frames which include averages of (2041-2060, 2061-2080, and 2081-2100). The predictions were done for two Representative Concentration RCP 4.5 and RCP 8.5 |
| Biodiversity data overview | **Observation type:** standardized monitoring  **Response data type:** presence/absence data |
| Type of predictors | Climatic |
| Conceptual model/hypotheses | A large body of scientific studies indicate that climate is one of the major drivers of the distribution of tree species at the continental scale. We exploited this correlation between species' current occurrence and climate to develop SDMs that predict the potential distribution of the target tree species. |
| Assumptions | We assumed that species are at pseudo-equilibrium with the environment. The source of the presence/absence data used in this study is largely from national forest inventories where tree individuals below a certain diameter at breast height are not recorded. We assume that this data collection procedure did not bias our occurrence data.  Since our occurrence dataset covers both the native and introduced range of the target species, which represents both the current and likely future climate of Europe, we assumed that the species retain their niches across space and time and the current occurrence-climate correlation remains stable when predicting the models for future climate. |
| SDM algorithms | **Algorithms:** We selected 10 modeling algorithms: GLM (Generalized Linear Models), GAM (Generalized Additive Models), GBM (Generalized Boosted regression Models), CTA (Classification Tree Analysis), ANN (Artificial Neural Networks), SRE (Surface Range Envelop or BIOCLIM), FDA (Flexible Discriminant Analysis), MARS (Multivariate Adaptive Regression Spline), RF (Random Forest for classification and regression), and MAXENT. Tsuruoka. These model algorithms were implemented through an ensemble model platform biomod2 (Thuiller et al., 2013).  **Model complexity:** The individual models were run using the standard default settings of biomod2, which are designed to balance model complexity and overfitting. See further details in the section “Model” detailed below.  **Ensembles:** The prediction of individual model algorithms were ensembled through biomod2 (Thuiller et al. 2013). See further details in the section “Model” detailed below. |
| Model workflow | The model workflow includes:   1. Data cleaning and presence-pseudoabsence generation 2. Variable selection 3. Model calibration 4. Ensemble prediction |
| Software | **Software:** All analyses were conducted using R version 3.3.2 (R Core Team, 2016). Packages used: biomod2 (Thuiller et al. 2013), Random Forest (Breiman, 2001).  **Climate data is available from**  WorldClimV2.0 (Fick and Hijmans 2017) |
| **DATA** | |
| Biodiversity data | **Taxon names:** *(See Table S1)*  **Ecological level:** *Species-level*  **Data source& sampling design**  Occurrence data (presence locations) of the target tree species in their native and introduced range in Europe was obtained from various sources such as National Forest Inventories, Global biodiversity facilities, private collection, and contributions from participating institutions in the EU COST Action [NNEXT](https://www.cost.eu/actions/FP1403/) (FP1403 - Non-native tree species for European forests - experiences, risks and opportunities). The sampling design varied in each of the data sources which were later harmonized through a data thinning approach (see Data filtering)  **Sample size.**  The dataset includes a total of 754, 413 occurrence records of the target species (i.e. presence locations).  **Data filtering:**  *Pseudoabsence generation*  For each species, we randomly selected 10,000 background points (Barbet‐Massin et al., 2012) using a 30-arc sec raster corresponding to the spatial resolution of the bioclimatic variables of worldclim2.0. This constituted the presence-pseudoabsence dataset.  *Data thinning and reducing spatial autocorrelation.*  The presence-pseudoabsence dataset was further thinned to reduce spatial autocorrelation by retaining only one occurrence (either presence or pseudoabsence) within each 30-arc sec raster cell of the bioclimatic variables from worldclim2.0.  After thinning, 734,076 presence records and 87700 pseudoabsences were retained for calibrating the SDMs with biomod2.  **Species occurrence data:**  This approach was used to generate pseudoabsence for all seven species.   \| **Species** \| **Presence** \| **Pseudoabsence** \| **Total** \| \| --- \| --- \| --- \| --- \| \| *Abies grandis* \| 74852 \| 9500 \| 84352 \| \| *Fraxinus pennsylvanica* \| 155693 \| 9500 \| 165193 \| \| *Juglans nigra* \| 55627 \| 9900 \| 65527 \| \| *Pinus contorta* \| 312066 \| 9800 \| 321866 \| \| *Pinus radiata* \| 5858 \| 9900 \| 15758 \| \| *Pseudotsuga menziesii* \| 23900 \| 9700 \| 33600 \| \| *Quercus rubra* \| 35328 \| 9800 \| 45128 \| \| *Robinia pseudoacacia* \| 69665 \| 9700 \| 79365 \| \| *Thuja plicata* \| 1086 \| 9900 \| 10986 \| \| **Total** \| 734076 \| 87700 \| 821776 \| |
| Data partitioning | The occurrence dataset for each target species was partitioned by splitting into 75% for model training and 25% for model evaluation. |
| Environmental predictors | **Predictor variables**  Environmental predictors were 19 biologically relevant climate variables comprising annual, seasonal, and monthly variables from Worldclim2.0(Fick and Hijmans 2017). See Table S2 in Supporting Information  From this list of 19 variables, a small subset of potential predictor variables was selected for each target species during the variable selection process.  **Data sources:**  **The spatial resolution of predictor data:** 30 arcsec which is roughly equivalent to 1x1km or lower depending on latitude.  **The temporal resolution of predictor variable:** Historic climate (1961-90) and three future time frames which include averages of (2041-2060, 2061-2080, and 2081-2100) for two future scenarios ssp245 and ssp585 which is equivalent to Representative Concentration Pathway scenarios RCP 4.5 and RCP 8.5 respectively. Future bioclimatic variables represent the mean of the 13 GMs presented in Worldclim2.0.  **Geographic projection:** WGS 84 (EPSG: 4326) |
| **Model** | |
| Variable selection and Multicollinearity | From the list of potential predictor variables (Table S2), the ones that explain most of the variation in the observed presence and absences of each species were selected with a recursive feature elimination approach (RFE) implemented within the Random forest algorithm (Breiman 2001b). Within the RFE approach, the variables were eliminated iteratively, starting from the full set of potential predictors (Table S2), and retaining only those variables that reduce the mean square error over random permutations of the same variable. The variables which were linearly correlated with other variables and had variance inflation factors VIF > 5 as suggested by (Booth et al., 1994)Click or tap here to enter text. were identified and the ones with the lower value according to the Akaike Information Criteria (AIC) (Akaike, 1974)Click or tap here to enter text. were retained for further model development.  This subset of bioclimatic climate variables was used as predictor variables for developing the ensemble species distribution models.   \| **Species** \| **Variable** \| Rank \| \| --- \| --- \| --- \| \| *Abies grandis* \| bio4 \| 1 \| \| *Abies grandis* \| bio7 \| 2 \| \| *Abies grandis* \| bio10 \| 3 \| \| *Abies grandis* \| bio19 \| 4 \| \| *Juglans nigra* \| bio12 \| 1 \| \| *Juglans nigra* \| bio19 \| 2 \| \| *Juglans nigra* \| bio10 \| 3 \| \| *Pinus contorta* \| bio10 \| 1 \| \| *Pinus contorta* \| bio19 \| 2 \| \| *Pinus contorta* \| bio11 \| 3 \| \| *Pinus contorta* \| bio12 \| 4 \| \| *Pseudotsuga menziesii* \| bio4 \| 1 \| \| *Pseudotsuga menziesii* \| bio10 \| 2 \| \| *Pseudotsuga menziesii* \| bio3 \| 3 \| \| *Pseudotsuga menziesii* \| bio5 \| 4 \| \| *Pinus radiata* \| bio4 \| 1 \| \| *Pinus radiata* \| bio11 \| 2 \| \| *Pinus radiata* \| bio12 \| 3 \| \| *Pinus radiata* \| bio1 \| 4 \| \| *Quercus rubra* \| bio4 \| 1 \| \| *Quercus rubra* \| bio1 \| 2 \| \| *Quercus rubra* \| bio10 \| 3 \| \| *Quercus rubra* \| bio7 \| 4 \| \| *Robinia pseudoacacia* \| bio4 \| 1 \| \| *Robinia pseudoacacia* \| bio10 \| 2 \| \| *Robinia pseudoacacia* \| bio17 \| 3 \| \| *Robinia pseudoacacia* \| bio5 \| 4 \| \| *Thuja plicata* \| bio4 \| 1 \| \| *Thuja plicata* \| bio1 \| 2 \| \| *Thuja plicata* \| bio7 \| 3 \| \| *Thuja plicata* \| bio11 \| 4 \| \| *Thuja plicata* \| bio6 \| 5 \| \| *Fraxinus pennsylvanica* \| bio4 \| 1 \| \| *Fraxinus pennsylvanica* \| bio16 \| 2 \| \| *Fraxinus pennsylvanica* \| bio8 \| 3 \| \| *Fraxinus pennsylvanica* \| bio1 \| 4 \| \| *Fraxinus pennsylvanica* \| bio7 \| 5 \| |
| Model settings | The models were run with the following settings of biomod2 as listed below  **Parameters for model calibration**   1. models = all models in biomod2 were used such as GLM, GBM, GAM, CTA, ANN, SRE, FDA, MARS, RF, and MAXENT.Tsuruoka, 2. CV.strateg = cross-validation selection strategy was set to random with 10 repetitions 3. CV.nb.rep = 10 repetitions 4. CV.perc = 0.75 (25% for model evaluation only). For model calibration full dataset was used 5. prevalence = 0.5, presences and absences will be weighted equally 6. metric.eval = Model evaluation metric TSS   **Parameter settings for individual model algorithms**  Generalised linear models (GLMs): GLMs were generated assuming a logistic link function and a binomial error distribution of the response variable without interactions.  Generalised Boosted Models (GBMs): GBMs were fitted with distribution = 'bernoulli' and a maximum of 25000 trees fitted to the data and with an interaction depth of 4  Generalised additive models (GAMs): GAMs were generated with default parameter in biomod2 with k (default 4): a smooth term cubic-smooth splines bounded by a degree of smoothness of four for each climatic predictor and family (binomial(link = 'logit'))  Classification tree analysis (CTA): CTA was carried out using method = 'class' for presence-absence data, parms = 'default', and 10 fold cross validation  Artificial neural networks (ANNs) with the following parameters   1. NbCV (default 5): nb of cross-validation to find best size and decay parameters; 2. size (default NULL): number of units in the hidden layer; 3. decay (default NULL): decay parameter will be optimized by cross-validation on model AUC i.e.. NbCv 4. rang (default 0.1) 5. maxit (default 200): maximum number of iterations.   Surface range envelopes (SREs): quant (default 0.025): quantile of 'extreme environmental variable' removed for selection of species envelops  Multivariate adaptive regression splines (MARS): MARS were fitted with default 0-level interactions between predictors.  Random Forests (RFs): The number of trees grown were set to 500 with mtry (default 'default'), nodesize (default 5) and maxnodes (default NULL)  MAXENT.Tsuruoka (Maxent) was used with the following default parameters in biomdo2   1. l1_regularizer (default 0.0): A numeric turning on L1 regularization and setting the regularization parameter. A value of 0 will disable L1 regularization 2. l2_regularizer (default 0.0): A numeric turning on L2 regularization and setting the regularization parameter. A value of 0 will disable L2 regularization 3. use_sgd (default FALSE): A logical indication that SGD parameter estimation should be used. Defaults to FALSE 4. set_heldout (default 0): An integer specifying the number of documents to hold out. Sets a held-out subset of your data to test against and prevent overfitting 5. verbose (default FALSE): A logical specifying whether to provide descriptive output about the training process |
| Model estimates | The models estimated the median ensemble probability of species occurrence. |
| Model ensemble | Predicted probabilities from the individual models for each target species were ensembled as a consensus model which combined the median probability over the selected models with the True Skill Statistics threshold (TSS > 0.7) (Allouche et al., 2006; Coetzee et al., 2009)Click or tap here to enter text..  **Parameters for model ensemble**   1. em.by= all ie. all models are combined into one ensemble 2. metric. select =a vector containing evaluation metric names ( TSS) to be used together with metric. 3. select. thresh = 0.7 for TSS   **Ensemble-models algorithms or the algorithms used for the ensemble were:**   1. EMmean : Mean of probabilities over the selected models. 2. EMmedian : Median of probabilities over the selected models. The median is less sensitive to outliers than the mean 3. EMcv : Coefficient of variation (sd/mean) of probabilities over the selected models. CV is a measure of uncertainty rather than a measure of the probability of occurrence. If the CV gets a high evaluation score, it means that the uncertainty is high where the species is observed |
| Threshold selection | and True Skill Statistics threshold (TSS > 0.7), a commonly used threshold for SDMs (Coetzee et al. 2009; Allouche, Tsoar, and Kadmon 2006) was used. |
| **Assessment** | |
| Model performance statistics | For each such model run as well as the final ensemble models for each target species, the model evaluation statistics were recorded. These statistics were true skill statistics (TSS), model sensitivity (the ability of the model to predict true presences), and model specificity (the ability of the model to predict the true absences). TSS takes into account both omission and commission errors and ranges also from −1 to +1, not being affected by prevalence as KAPPA (Allouche, Tsoar, and Kadmon 2006). TSS values ranging from 0.2 to 0.5 were considered poor, from 0.6 to 0.8 useful, and values larger than 0.8 were good to excellent (Coetzee et al. 2009).  Statistics for evaluation for each of the models used to develop the ensemble SDM for the seven tree species. MAXENT   \| **Species** \| **Criteria** \| **Testing.data** \| **Sensitivity** \| **Specificity** \| **Model** \| \| --- \| --- \| --- \| --- \| --- \| --- \| \| *Abies grandis* \| TSS \| 1.0 \| 99.3 \| 99.7 \| GLM \| \| *Abies grandis* \| TSS \| 1.0 \| 99.1 \| 98.6 \| GAM \| \| *Abies grandis* \| TSS \| 1.0 \| 98.9 \| 98.5 \| GBM \| \| *Abies grandis* \| TSS \| 1.0 \| 98.6 \| 98.6 \| CTA \| \| *Abies grandis* \| TSS \| 0.9 \| 98.4 \| 95.1 \| ANN \| \| *Abies grandis* \| TSS \| 1.0 \| 99.9 \| 100.0 \| RF \| \| *Abies grandis* \| TSS \| 1.0 \| 99.0 \| 98.9 \| FDA \| \| *Abies grandis* \| TSS \| 1.0 \| 98.2 \| 98.6 \| MARS \| \| *Abies grandis* \| TSS \| 1.0 \| 98.0 \| 97.0 \| Maxent \| \| *Fraxinus pennsylvanica* \| TSS \| 1.0 \| 99.0 \| 99.5 \| GLM \| \| *Fraxinus pennsylvanica* \| TSS \| 1.0 \| 98.2 \| 98.6 \| GAM \| \| *Fraxinus pennsylvanica* \| TSS \| 1.0 \| 98.8 \| 99.1 \| GBM \| \| *Fraxinus pennsylvanica* \| TSS \| 1.0 \| 98.6 \| 99.0 \| CTA \| \| *Fraxinus pennsylvanica* \| TSS \| 0.9 \| 93.5 \| 98.2 \| ANN \| \| *Fraxinus pennsylvanica* \| TSS \| 1.0 \| 100.0 \| 100.0 \| RF \| \| *Fraxinus pennsylvanica* \| TSS \| 1.0 \| 98.7 \| 99.3 \| FDA \| \| *Fraxinus pennsylvanica* \| TSS \| 1.0 \| 99.1 \| 99.1 \| MARS \| \| *Fraxinus pennsylvanica* \| TSS \| 0.9 \| 95.3 \| 98.0 \| MAXENT \| \| *Juglans nigra* \| TSS \| 1.0 \| 99.3 \| 98.7 \| GLM \| \| *Juglans nigra* \| TSS \| 1.0 \| 98.4 \| 99.3 \| GAM \| \| *Juglans nigra* \| TSS \| 1.0 \| 99.4 \| 99.9 \| GBM \| \| *Juglans nigra* \| TSS \| 1.0 \| 99.2 \| 99.6 \| CTA \| \| *Juglans nigra* \| TSS \| 0.9 \| 92.2 \| 93.7 \| ANN \| \| *Juglans nigra* \| TSS \| 1.0 \| 100.0 \| 100.0 \| RF \| \| *Juglans nigra* \| TSS \| 1.0 \| 100.0 \| 95.6 \| FDA \| \| *Juglans nigra* \| TSS \| 1.0 \| 98.7 \| 98.6 \| MARS \| \| *Juglans nigra* \| TSS \| 1.0 \| 98.7 \| 96.7 \| MAXENT \| \| *Pinus contorta* \| TSS \| 1.0 \| 98.5 \| 99.0 \| GLM \| \| *Pinus contorta* \| TSS \| 1.0 \| 98.2 \| 97.9 \| GAM \| \| *Pinus contorta* \| TSS \| 1.0 \| 97.9 \| 98.5 \| GBM \| \| *Pinus contorta* \| TSS \| 1.0 \| 98.5 \| 98.1 \| CTA \| \| *Pinus contorta* \| TSS \| 1.0 \| 100.0 \| 100.0 \| RF \| \| *Pinus contorta* \| TSS \| 1.0 \| 98.5 \| 99.0 \| FDA \| \| *Pinus contorta* \| TSS \| 1.0 \| 98.9 \| 99.0 \| MARS \| \| *Pinus contorta* \| TSS \| 0.8 \| 88.3 \| 93.2 \| MAXENT \| \| *Pseudotsuga menziesii* \| TSS \| 1.0 \| 99.1 \| 98.7 \| GLM \| \| *Pseudotsuga menziesii* \| TSS \| 1.0 \| 98.2 \| 98.5 \| GAM \| \| *Pseudotsuga menziesii* \| TSS \| 1.0 \| 98.8 \| 98.9 \| GBM \| \| *Pseudotsuga menziesii* \| TSS \| 1.0 \| 98.2 \| 99.3 \| CTA \| \| *Pseudotsuga menziesii* \| TSS \| 0.9 \| 97.7 \| 96.9 \| ANN \| \| *Pseudotsuga menziesii* \| TSS \| 1.0 \| 100.0 \| 100.0 \| RF \| \| *Pseudotsuga menziesii* \| TSS \| 1.0 \| 98.4 \| 98.8 \| MARS \| \| *Pseudotsuga menziesii* \| TSS \| 0.9 \| 97.0 \| 97.0 \| MAXENT \| \| *Pinus radiata* \| TSS \| 1.0 \| 99.2 \| 96.7 \| GLM \| \| *Pinus radiata* \| TSS \| 1.0 \| 99.3 \| 99.7 \| GAM \| \| *Pinus radiata* \| TSS \| 1.0 \| 99.9 \| 99.6 \| GBM \| \| *Pinus radiata* \| TSS \| 1.0 \| 99.7 \| 98.0 \| CTA \| \| *Pinus radiata* \| TSS \| 1.0 \| 99.7 \| 99.1 \| ANN \| \| *Pinus radiata* \| TSS \| 1.0 \| 100.0 \| 100.0 \| RF \| \| *Pinus radiata* \| TSS \| 1.0 \| 99.9 \| 98.7 \| FDA \| \| *Pinus radiata* \| TSS \| 1.0 \| 100.0 \| 100.0 \| MARS \| \| *Pinus radiata* \| TSS \| 1.0 \| 99.0 \| 98.6 \| MAXENT \| \| *Quercus rubra* \| TSS \| 1.0 \| 99.3 \| 99.5 \| GLM \| \| *Quercus rubra* \| TSS \| 1.0 \| 98.2 \| 99.4 \| GAM \| \| *Quercus rubra* \| TSS \| 1.0 \| 98.7 \| 99.0 \| GBM \| \| *Quercus rubra* \| TSS \| 1.0 \| 98.3 \| 99.4 \| CTA \| \| *Quercus rubra* \| TSS \| 1.0 \| 97.7 \| 97.6 \| ANN \| \| *Quercus rubra* \| TSS \| 1.0 \| 100.0 \| 100.0 \| RF \| \| *Quercus rubra* \| TSS \| 1.0 \| 99.2 \| 98.2 \| FDA \| \| *Quercus rubra* \| TSS \| 1.0 \| 98.7 \| 98.6 \| MARS \| \| *Quercus rubra* \| TSS \| 0.9 \| 97.1 \| 97.3 \| MAXENT \| \| *Robinia pseudoacacia* \| TSS \| 1.0 \| 98.3 \| 98.9 \| GLM \| \| *Robinia pseudoacacia* \| TSS \| 0.9 \| 97.2 \| 97.4 \| GAM \| \| *Robinia pseudoacacia* \| TSS \| 1.0 \| 98.3 \| 97.9 \| GBM \| \| *Robinia pseudoacacia* \| TSS \| 1.0 \| 97.8 \| 97.7 \| CTA \| \| *Robinia pseudoacacia* \| TSS \| 0.9 \| 97.8 \| 90.5 \| ANN \| \| *Robinia pseudoacacia* \| TSS \| 1.0 \| 100.0 \| 100.0 \| RF \| \| *Robinia pseudoacacia* \| TSS \| 0.9 \| 97.4 \| 97.3 \| MARS \| \| *Robinia pseudoacacia* \| TSS \| 0.9 \| 95.6 \| 94.5 \| MAXENT \| \| *Thuja plicata* \| TSS \| 1.0 \| 99.3 \| 98.6 \| GLM \| \| *Thuja plicata* \| TSS \| 1.0 \| 98.8 \| 98.4 \| GAM \| \| *Thuja plicata* \| TSS \| 1.0 \| 98.8 \| 99.8 \| GBM \| \| *Thuja plicata* \| TSS \| 1.0 \| 99.1 \| 98.0 \| CTA \| \| *Thuja plicata* \| TSS \| 0.9 \| 97.0 \| 96.8 \| ANN \| \| *Thuja plicata* \| TSS \| 1.0 \| 100.0 \| 100.0 \| RF \| \| *Thuja plicata* \| TSS \| 1.0 \| 99.3 \| 99.0 \| FDA \| \| *Thuja plicata* \| TSS \| 1.0 \| 99.5 \| 99.8 \| MARS \| \| *Thuja plicata* \| TSS \| 0.9 \| 97.2 \| 96.8 \| MAXENT \|   Statistics for evaluation for the ensemble SDM for the target species   \|  \| Model evaluation \| \| \| \| \| \| --- \| --- \| --- \| --- \| --- \| --- \| \| Species \| Criteria \| Testing data \| Evaluation data \| Sensitivity \| Specificity \| \| *Abies grandis* \| TSS \| 0.97 \| 0.99 \| 98.84 \| 98.32 \| \| *Fraxinus pennsylvanica* \| TSS \| 0.97 \| 0.96 \| 97.92 \| 98.97 \| \| *Juglans nigra* \| TSS \| 0.96 \| 0.97 \| 98.42 \| 98.00 \| \| *Pinus contorta* \| TSS \| 0.95 \| 0.97 \| 97.36 \| 98.08 \| \| *Pseudotsuga menziesii* \| TSS \| 0.97 \| 0.95 \| 98.42 \| 98.49 \| \| *Pinus radiata* \| TSS \| 0.99 \| 0.96 \| 99.63 \| 98.94 \| \| *Quercus rubra* \| TSS \| 0.97 \| 0.98 \| 98.58 \| 98.77 \| \| *Robinia pseudoacacia* \| TSS \| 0.95 \| 0.99 \| 97.81 \| 96.75 \| \| *Thuja plicata* \| TSS \| 0.97 \| 0.96 \| 98.76 \| 98.58 \| |
| **Prediction** | |
| Prediction output | Predicted probabilities from the individual models and target species were ensembled as a consensus model which combined the median probability over the selected models with True Skill Statistics threshold (TSS > 0.7) (Coetzee et al. 2009; Allouche, Tsoar, and Kadmon 2006). The median was chosen because it is known to be less sensitive to outliers than the mean. The estimated ensemble model predictions were presented as GeoTIFF rasters |


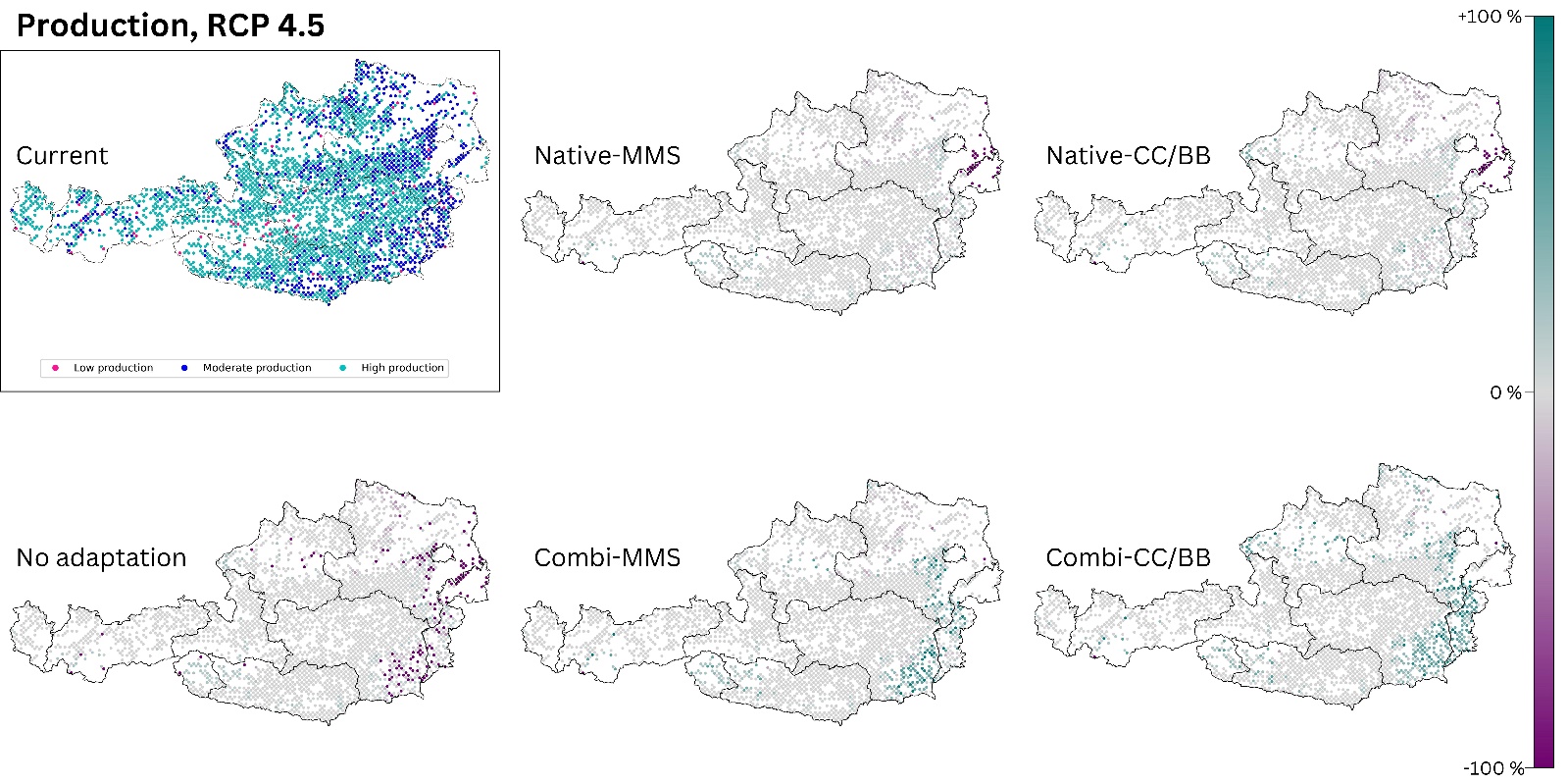


Figure S4: The production output of today’s climate is compared with those of the five species change scenarios for future climate (2081-2100, RCP 4.5). The section titled 'current' displays the present status of the analysed ecosystem service. The effects of the 5 species change scenarios are also shown. Purple dots indicate a negative change compared to the current state, and turquoise dots indicate a positive change. (Native): only native species are used; (Combi): Native and non-native species are used; (MSS): a tree species that fails is replaced by the most climatically suitable species, either coniferous or broadleaved; (CC/BB): coniferous species are primarily replaced by coniferous species and broadleaved species are primarily replaced by broadleaved species.


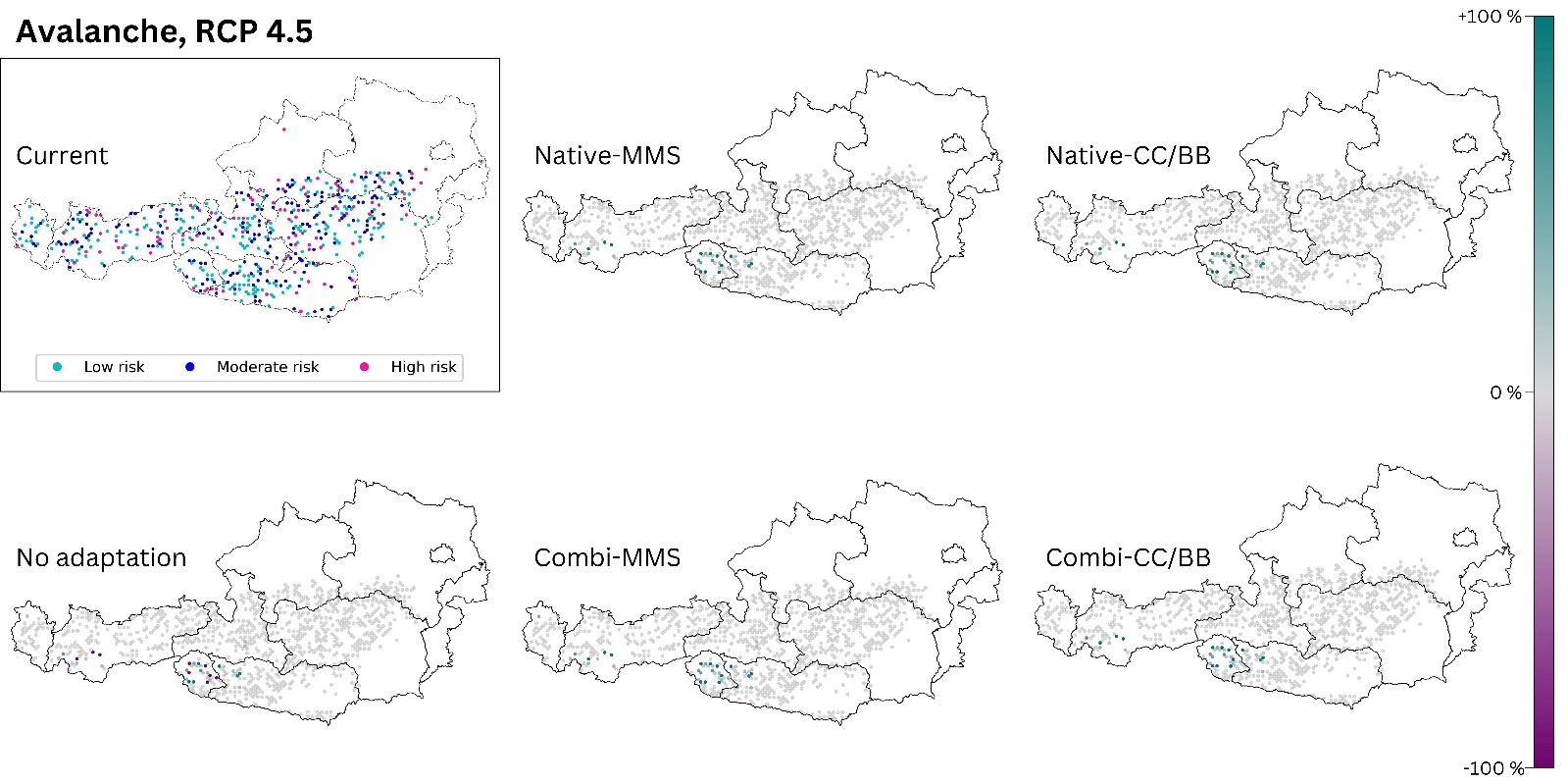


Figure S5: The avalanche risk of today’s climate is compared with those of the five species change scenarios for future climate (2081-2100, RCP 4.5). The section titled 'current' displays the present status of the analysed ecosystem service. The effects of the 5 species change scenarios are also shown. Purple dots indicate a negative change compared to the current state, and turquoise dots indicate a positive change.  (Native): only native species are used; (Combi): Native and non-native species are used; (MSS): a tree species that fails is replaced by the most climatically suitable species, either coniferous or broadleaved; (CC/BB): coniferous species are primarily replaced by coniferous species and broadleaved species are primarily replaced by broadleaved species. The number of plots was limited to those where avalanches are possible (see methods section). This restriction resulted in blank areas on the map.


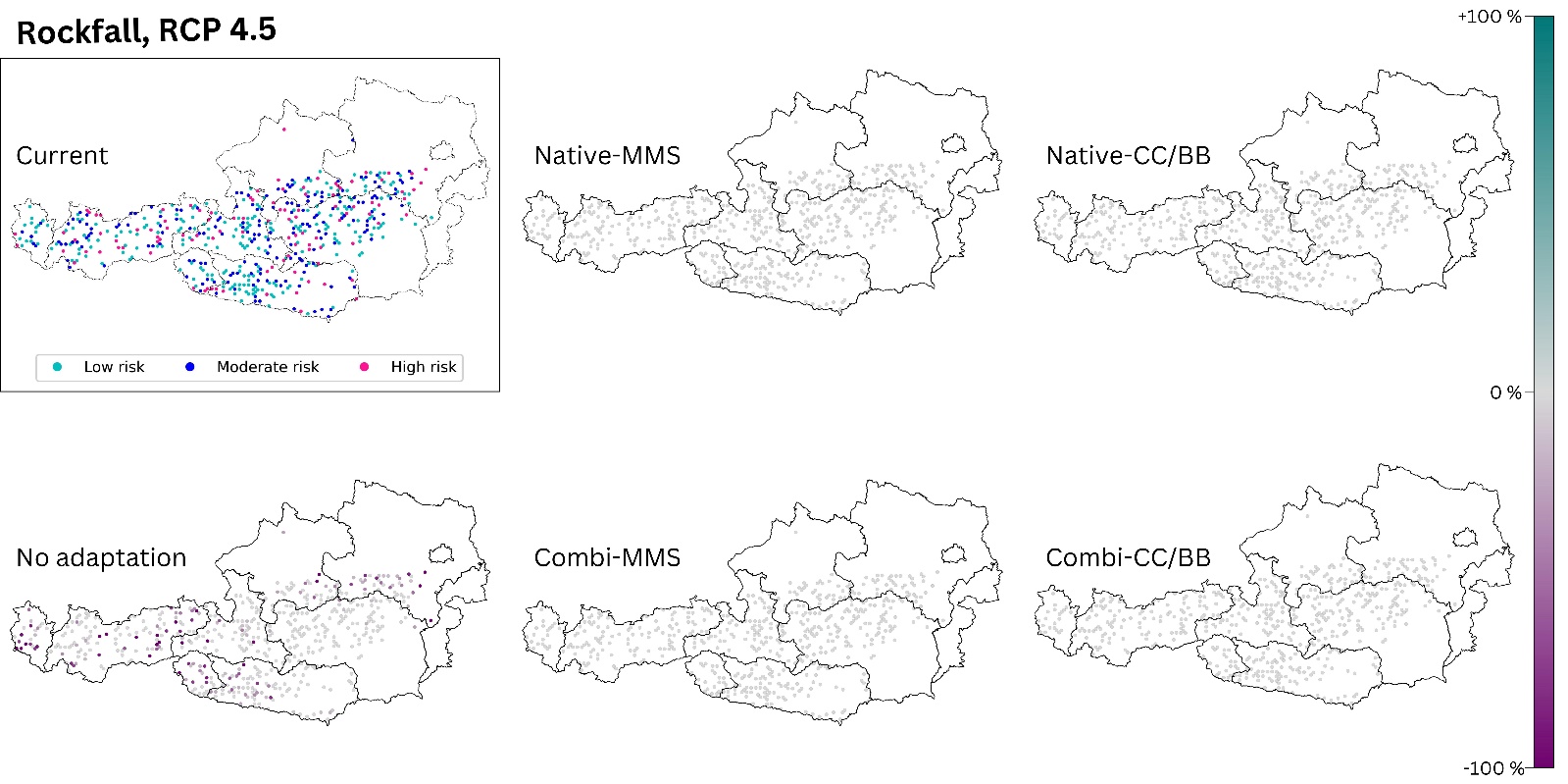


Figure S6: The rockfall risk of today’s climate is compared with those of the five species change scenarios for future climate (2081-2100, RCP 4.5). The section titled 'current' displays the present status of the analysed ecosystem service. The effects of the 5 species change scenarios are also shown. Purple dots indicate a negative change compared to the current state, and turquoise dots indicate a positive change.  (Native): only native species are used; (Combi): Native and non-native species are used; (MSS): a tree species that fails is replaced by the most climatically suitable species, either coniferous or broadleaved; (CC/BB): coniferous species are primarily replaced by coniferous species and broadleaved species are primarily replaced by broadleaved species. The number of plots was restricted to those where rockfall has already occurred (see methods), resulting in blank areas on the map.

**References**

Akaike, H., 1974. A new look at the statistical model identification. IEEE Trans. Autom. Control 19, 716–723. https://doi.org/10.1109/TAC.1974.1100705

Allouche, O., Tsoar, A., Kadmon, R., 2006. Assessing the accuracy of species distribution models: prevalence, kappa and the true skill statistic (TSS). J. Appl. Ecol. 43, 1223–1232. https://doi.org/10.1111/j.1365-2664.2006.01214.x

Antos, J.A., Filipescu, C.N., Negrave, R.W., 2016. Ecology of western redcedar (Thuja plicata): Implications for management of a high-value multiple-use resource. For. Ecol. Manag. 375, 211–222. https://doi.org/10.1016/j.foreco.2016.05.043

Barbet‐Massin, M., Jiguet, F., Albert, C.H., Thuiller, W., 2012. Selecting pseudo‐absences for species distribution models: how, where and how many? Methods Ecol. Evol. 3, 327–338. https://doi.org/10.1111/j.2041-210X.2011.00172.x

Bauhus, J., Bindewald, A., Michiels, H.-G., 2017. Douglasie – Potenziale, Risiken und Invasivitätsbewertung. Allg. Forstz.- Wald 34–36.

Bindewald, A., Michiels, H.-G., 2016. Quantifying invasiveness of Douglas fir on the basis of natural regeneration in south-western Germany, in: Introduced Tree Species in European Forests: Opportunities and Challenges. pp. 330–343.

Booth, G.D., Niccolucci, M.J., Schuster, E.G., 1994. Identifying proxy sets in multiple linear regression: an aid to better coefficient interpretation. USDA For. Serv. Intermt. Res. Stn. Res. Pap.

Brauer, A., 2015. Quercus rubra, in: Stimm, B., Roloff, A., Lang, U.M., Weisgerber, H. (Eds.), Enzyklopädie der Holzgewächse: Handbuch und Atlas der Dendrologie. Wiley-VCH Verlag GmbH & Co. KGaA, Weinheim, Germany, pp. 1–26. https://doi.org/10.1002/9783527678518.ehg2014005

Breiman, L., 2001. [No title found]. Mach. Learn. 45, 5–32. https://doi.org/10.1023/A:1010933404324

Coetzee, B.W.T., Robertson, M.P., Erasmus, B.F.N., Van Rensburg, B.J., Thuiller, W., 2009. Ensemble models predict Important Bird Areas in southern Africa will become less effective for conserving endemic birds under climate change. Glob. Ecol. Biogeogr. 18, 701–710. https://doi.org/10.1111/j.1466-8238.2009.00485.x

Davis, K.T., Callaway, R.M., Fajardo, A., Pauchard, A., Nuñez, M.A., Brooker, R.W., Maxwell, B.D., Dimarco, R.D., Peltzer, D.A., Mason, B., Ruotsalainen, S., McIntosh, A.C.S., Pakeman, R.J., Smith, A.L., Gundale, M.J., 2019. Severity of impacts of an introduced species corresponds with regional eco‐evolutionary experience. Ecography 42, 12–22. https://doi.org/10.1111/ecog.04014

Drescher, A., Prots, B., 2016. Fraxinus pennsylvanica-an invasive tree species in Middle Europe: case studies from the Danube basin.

Dreßel, R., Jäger, E.J., 2002. Beiträge zur Biologie der Gefäßpflanzen des herzynischen Raumes. 5. Quercus rubra L.(Roteiche): Lebensgeschichte und agriophytische Ausbreitung im Nationalpark Sächsische Schweiz. Hercynia-Ökol. Umw. Mitteleur. 35, 37–64.

Engelmark, O., Sjöberg, K., Andersson, B., Rosvall, O., Ågren, G.I., Baker, W.L., Barklund, P., Björkman, C., Despain, D.G., Elfving, B., Ennos, R.A., Karlman, M., Knecht, M.F., Knight, D.H., Ledgard, N.J., Lindelöw, Å., Nilsson, C., Peterken, G.F., Sörlin, S., Sykes, M.T., 2001. Ecological effects and management aspects of an exotic tree species: the case of lodgepole pine in Sweden. For. Ecol. Manag. 141, 3–13. https://doi.org/10.1016/S0378-1127(00)00498-9

Essl, F., Rabitsch, W., 2002. Neobiota in Österreich. Umweltbundesamt, Wien.

Fanal, A., Mahy, G., Fayolle, A., Monty, A., 2021. Arboreta reveal the invasive potential of several conifer species in the temperate forests of western Europe. NeoBiota 64, 23–42. https://doi.org/10.3897/neobiota.64.56027

Fick, S.E., Hijmans, R.J., 2017. WorldClim 2: new 1‐km spatial resolution climate surfaces for global land areas. Int. J. Climatol. 37, 4302–4315. https://doi.org/10.1002/joc.5086

Foiles, M.W., Graham, R.T., Olson Jr, D.F., 1990. Abies grandis (Dougl. ex D. Don.) Lindl. Silv. N. Am. 1, 52–9.

Hermann, R.K., 2014. Pseudotsuga menziesii, in: Stimm, A., Roloff, U.M., Weisgerber, H. (Eds.), Enzyklopädie der Holzgewächse: Handbuch und Atlas der Dendrologie. John Wiley & Sons, Ltd. https://doi.org/10.1002/9783527678518.ehg2000045

Huntley, J.C., 1990. Robinia pseudoacacia L. black locust. Silv. N. Am. ‐ Harwoods 2, 755–761.

Lindenmayer, D.B., McCarthy, M.A., 2001. The spatial distribution of non-native plant invaders in a pine–eucalypt landscape mosaic in south-eastern Australia. Biol. Conserv. 102, 77–87. https://doi.org/10.1016/S0006-3207(01)00089-1

Marinšek, A., Bindewald, A., Kraxner, F., La Porta, N., Meisel, P., Lapin, K., 2022. Management of non-native tree species in forests of the Alpine space.

McDonald, P.M., Laacke, R.J., 1990. Pinus radiata D. Don, Monterey pine. Silv. N. Am. 1, 433–441.

Nicolescu, V.-N., Rédei, K., Vor, T., Bastien, J.-C., Brus, R., Benčať, T., Đodan, M., Cvjetkovic, B., Andrašev, S., La Porta, N., Lavnyy, V., Petkova, K., Perić, S., Bartlett, D., Hernea, C., Pástor, M., Mataruga, M., Podrázský, V., Sfeclă, V., Štefančik, I., 2020. A review of black walnut (Juglans nigra L.) ecology and management in Europe. Trees 34, 1087–1112. https://doi.org/10.1007/s00468-020-01988-7

Richardson, D.M., Rejmánek, M., 2004. Conifers as invasive aliens: a global survey and predictive framework: Global invasiveness of conifers. Divers. Distrib. 10, 321–331. https://doi.org/10.1111/j.1366-9516.2004.00096.x

Rietveld, W.J., 1983. Allelopathic effects of juglone on germination and growth of several herbaceous and woody species. J. Chem. Ecol. 9, 295–308. https://doi.org/10.1007/BF00988047

Ruetz, W., 2014. Abies grandis, in: Enzyklopädie der Holzgewächse: Handbuch und Atlas der Dendrologie. John Wiley & Sons, Ltd, pp. 1–10. https://doi.org/10.1002/9783527678518.ehg2011000

Schaarschmidt, H., 2004. Juglans nigra, in: Stimm, B., Roloff, A., Lang, U.M., Weisgerber, H. (Eds.), Enzyklopädie der Holzgewächse: Handbuch und Atlas der Dendrologie. Wiley-VCH Verlag GmbH & Co. KGaA, Weinheim, Germany, pp. 1–16. https://doi.org/10.1002/9783527678518.ehg2012004

Schmiedel, D., 2007. Fraxinus pennsylvanica, in: Schütt, P., Roloff, A., Weisgerber, H., Lang, U.M., Stimm, B. (Eds.), Enzyklopädie der Holzgewächse: Handbuch und Atlas der Dendrologie. Wiley-VCH, Weinheim.

Schütt, P., 2014. Robinia pseudoacacia LINNÉ, 1753, in: Schütt, P., Roloff, A., Weisgerber, H., Lang, U.M., Stimm, B. (Eds.), Enzyklopädie der Holzgewächse: Handbuch und Atlas der Dendrologie. Wiley-VCH, Weinheim.

Spellmann, H., Brang, P., Hein, S., Geb, M., 2015. 4. Baumartenportraits Baumartenportraits - Große Küstentanne, in: Potenziale und Risiken eingeführter Baumarten. Baumartenportraits mit naturschutzfachlicher Bewertung.

Stephan, B.R., 2014. Pinus contorta, in: Roloff, A., Schütt, P., Weisgerber, H., Lang, U.M., Stimm, B. (Eds.), Enzyklopädie der Holzgewächse: Handbuch und Atlas der Dendrologie, Ecomed Biowissenschaften. ecomed, Landsberg am Lech.

Thuiller, W., Georges, D., Engler, R., Breiner, F., 2013. biomod2: Ensemble platform for species distribution modeling. R Package Version 3, r539.

Vor, T., Spellmann, H., Bolte, A., Ammer, C., Bartsch, N. (Eds.), 2015. Potenziale und Risiken eingeführter Baumarten: Baumartenportraits mit naturschutzfachlicher Bewertung, Göttinger Forstwissenschaften. Universitätsverlag Göttingen, Göttingen.

Watson, A., O’loughlin, C., 1990. Structural root morphology and biomass of three age classes of Pinus radiata. N. Z. J. For. Sci. 20, 97–110.

Wickler, E.F., 2014. Thuja plicata DONNex D. DON, 1824, in: Roloff, A., Schütt, P., Weisgerber, H., Lang, U.M., Stimm, B. (Eds.), Enzyklopädie der Holzgewächse: Handbuch und Atlas der Dendrologie, Ecomed Biowissenschaften. ecomed, Landsberg am Lech.

Williams, M.C., Wardle, G.M., 2007. Pinus radiata invasion in Australia: Identifying key knowledge gaps and research directions. Austral Ecol. 32, 721–739. https://doi.org/10.1111/j.1442-9993.2007.01760.x

Zurell, D., Franklin, J., König, C., Bouchet, P.J., Dormann, C.F., Elith, J., Fandos, G., Feng, X., Guillera‐Arroita, G., Guisan, A., Lahoz‐Monfort, J.J., Leitão, P.J., Park, D.S., Peterson, A.T., Rapacciuolo, G., Schmatz, D.R., Schröder, B., Serra‐Diaz, J.M., Thuiller, W., Yates, K.L., Zimmermann, N.E., Merow, C., 2020. A standard protocol for reporting species distribution models. Ecography 43, 1261–1277. https://doi.org/10.1111/ecog.04960

5Fi
